# IL-1β^+^ lung-resident macrophages mediate endothelial dysfunction and acute lung injury in sepsis through immune-metabolic crosstalk

**DOI:** 10.1038/s41420-025-02868-0

**Published:** 2025-12-08

**Authors:** Yang Dong, Tianyuan Li, Bei Fang, Dingde Long, Ying Tian, Huan Fu

**Affiliations:** Department of Anesthesiology, The First Affiliated Hospital, Jiangxi Medical College, Nanchang University, Nanchang, China

**Keywords:** Respiratory distress syndrome, Immune cell death

## Abstract

Sepsis-induced acute lung injury (ALI) involves a complex interplay between immune cells and the pulmonary endothelium. However, the molecular regulators that coordinate this interaction remain poorly defined. In a murine sepsis model, we identified a subset of lung-resident macrophages characterized by robust IL-1β expression as pivotal contributors to lung damage. Single-cell RNA sequencing (scRNA-seq) delineated a distinct IL-1β⁺ macrophage population with pronounced pro-inflammatory transcriptional features and enhanced endothelial communication. These macrophages exhibited intensified ligand–receptor interactions with pulmonary endothelial cells, corresponding with elevated vascular leakage and histopathological evidence of injury. Immunoassays, Western blotting, and histopathology confirmed IL-1β upregulation during lung injury. Furthermore, metabolomics and in vitro co-culture experiments demonstrated that IL-1β impairs endothelial integrity and modulates metabolic activity. This study reveals a novel immune-metabolic axis whereby IL-1β^+^ macrophages orchestrate endothelial dysfunction and tissue injury in sepsis. Our findings highlight IL-1β as a potential therapeutic target for mitigating ALI in septic patients.

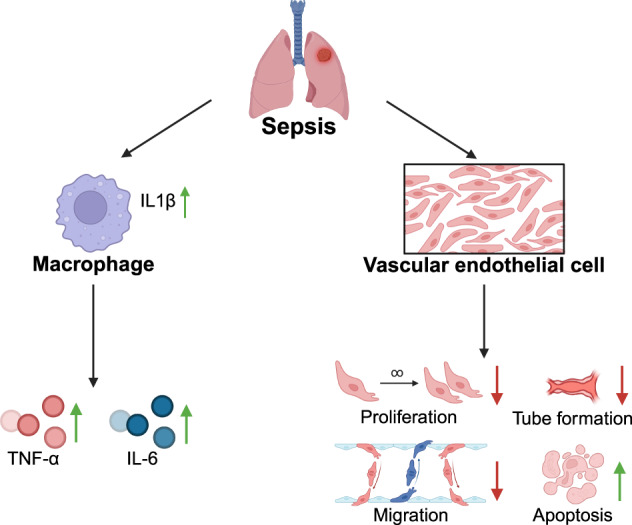

## Introduction

Sepsis, a life-threatening systemic inflammatory response commonly initiated by infection, remains a major global health challenge due to its rapid progression to multiorgan failure and high mortality [1, 2]. Acute lung injury (ALI) represents one of the most severe complications of sepsis, often leading to multi-organ damage and critically affecting patient survival [3, 4]. Pathological changes in the lungs during sepsis, such as alveolar congestion, edema, and inflammatory cell infiltration, result in substantial respiratory dysfunction. Since the lungs are directly exposed to infections and inflammatory responses, understanding the mechanisms underlying sepsis-induced lung injury is essential for developing effective treatments.

Sepsis-induced lung injury involves multiple cell types, including lung-resident macrophages, endothelial cells, and T cells [5]. These cells exacerbate tissue damage by releasing cytokines and chemokines that drive inflammation [6]. IL-1β, a critical pro-inflammatory mediator, plays a central role by activating lung-resident macrophages and influencing the permeability and function of pulmonary endothelial cells. IL-1β stimulates macrophages to release cytokines such as TNF-α, further triggering endothelial dysfunction and vascular permeability. This positive feedback loop intensifies inflammation, contributing to lung tissue damage [7, 8].

Traditional biochemical methods have provided valuable information about cytokine-mediated inflammation but failed to uncover detailed intercellular interactions at the cellular level. Single-cell RNA sequencing (scRNA-seq) enables high-throughput gene expression analysis in individual cells, providing insights into cellular heterogeneity and dynamic communication [9, 10]. This technology can identify subtle changes in cell states, map intercellular communication pathways, and reveal the molecular mechanisms driving pathological processes.

This study employed scRNA-seq to systematically explore for the first time the interactions and molecular mechanisms between IL-1β^+^ lung-resident macrophages and pulmonary endothelial cells in sepsis-induced lung injury. Utilizing a sepsis mouse model, we meticulously documented physiological changes under pathological conditions and observed lung tissue pathology using hematoxylin and eosin (H&E) staining and various molecular biology techniques. Further, single cells were isolated from both normal and injured lung tissues for RNA sequencing. We detailed the variations in different cell types during sepsis through dimensionality reduction and cluster analysis. Additionally, tools like CellChat were used to analyze intercellular signaling, revealing the pivotal role of IL-1β in regulating cell interactions.

The p38 mitogen-activated protein kinase (MAPK) pathway is a crucial regulatory axis for cellular stress responses and inflammatory signal transduction, playing a significant role in lung injury caused by sepsis. This pathway is typically activated by cytokines like TNF-α and IL-1β or external stimuli like LPS. Once activated, it phosphorylates downstream effectors, regulating the expression of various inflammation-related genes and cellular functions [11]. Studies have demonstrated that excessive activation of the p38 MAPK pathway is closely associated with endothelial cell dysfunction, including inhibited proliferation, reduced migratory and repair capacities, and increased apoptosis. These changes exacerbate the disruption of the pulmonary vascular barrier, leading to pulmonary edema and the spread of inflammation [12]. In septic conditions, macrophages act as major sources of inflammatory mediators, and their secretion of IL-1β and TNF-α potently stimulates p38 MAPK signaling in endothelial cells, thereby aggravating vascular injury. Pharmacologic inhibition of p38 MAPK alleviates endothelial damage, restores reparative function, and markedly attenuates lung injury in experimental sepsis models [13, 14]. These findings highlight the pivotal role of the p38 MAPK pathway in the development of sepsis-induced lung injury, underscoring its potential as a therapeutic target.

The primary objective of this study was to delve into the molecular interactions between IL-1β^+^ lung-resident macrophages and pulmonary endothelial cells during sepsis-induced lung injury, using scRNA-seq. By revealing the patterns of interaction and communication among these cells under pathological conditions, we aimed to enhance our understanding of the cellular and molecular mechanisms driving lung injury in sepsis. These insights deepen our comprehension of the complex biological processes (BP) involved in sepsis-induced lung injury and lay a foundation for the development of precision therapies. For instance, targeting the interactions between IL-1β^+^ macrophages and pulmonary endothelial cells could lead to developing novel therapeutics that effectively alleviate or prevent lung injury caused by sepsis. Furthermore, the findings from this study provide a crucial methodological and theoretical basis for using single-cell technologies to investigate other inflammatory diseases. Collectively, this research not only enriches the current body of knowledge on sepsis biology but also holds translational potential for improving therapeutic efficacy and patient prognosis.

## Results

### Successful establishment of a sepsis-induced lung injury mouse model

**Fig. 1:**
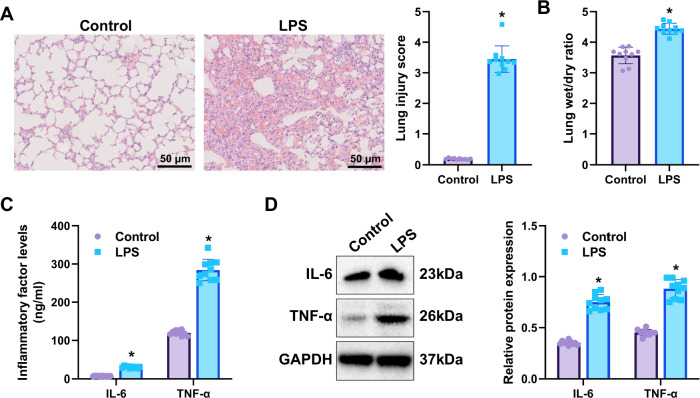
Increased expression of inflammatory markers in the LPS-induced sepsis mouse model. **A** H&E-stained lung tissue sections showing significant inflammatory cell infiltration, lung-resident structural disruption, and interstitial edema in the lung tissue of LPS-treated mice (scale bar: 50 µm); **B** Wet/dry weight ratios of lung tissue in each group of mice; **C** ELISA analysis of IL-6 and TNF-α levels in the serum of each group of mice; **D** WB analysis of IL-6 and TNF-α protein expression in the lung tissue of each group of mice. *indicates *p* < 0.05 compared to the Control group, with 10 mice per group.

All mice were randomly assigned to either the Control or experimental group. Sepsis-induced ALI was established in the experimental group by intraperitoneal administration of lipopolysaccharide (LPS). After 24 hours, lung tissues were harvested for histopathological evaluation. Hematoxylin and eosin (H&E) staining revealed pronounced inflammatory cell infiltration, disruption of alveolar architecture, and marked interstitial edema in LPS-treated mice, as quantified by lung injury scores (Fig. 1A). To further assess pulmonary edema, the wet-to-dry (W/D) weight ratio of lung tissue was determined, showing a significant elevation in the LPS group compared with controls (Fig. 1B). These findings indicate that LPS treatment successfully induced a sepsis-induced lung injury model in mice.

Enzyme-linked immunosorbent assay (ELISA) and Western blotting (WB) were performed to quantify the inflammatory mediators IL-6 and TNF-α in both serum and lung tissue, thereby verifying the successful induction of the sepsis-induced lung injury model. ELISA results revealed markedly higher serum concentrations of IL-6 and TNF-α in LPS-treated mice compared with controls (Fig. 1C). Consistently, WB analysis demonstrated elevated IL-6 and TNF-α protein expression in lung tissues from the LPS group relative to the Control group (Fig. 1D).

These data confirm that LPS administration triggered a robust inflammatory response, validating the successful establishment of the sepsis-induced acute lung injury model.

### scRNA-seq reveals communication activation between pulmonary macrophages and endothelial cells in sepsis

To investigate cellular changes in lung tissues from normal and sepsis-injured mice, we successfully extracted single-cell suspensions from their lung tissues and performed single-cell RNA sequencing (scRNA-seq) using the 10x Genomics platform. After integrating and filtering the sample data using the Seurat package, we obtained an expression matrix comprising 15,239 genes and 25,729 cells (Fig. S1A, B). Correlation analysis demonstrated a negative relationship between sequencing depth (nCount_RNA) and mitochondrial gene percentage (percent.mt) (r = –0.14) and a strong positive correlation between nCount_RNA and nFeature_RNA (r = 0.91), confirming the high quality of the filtered dataset (Fig. S1C).

**Fig. 2:**
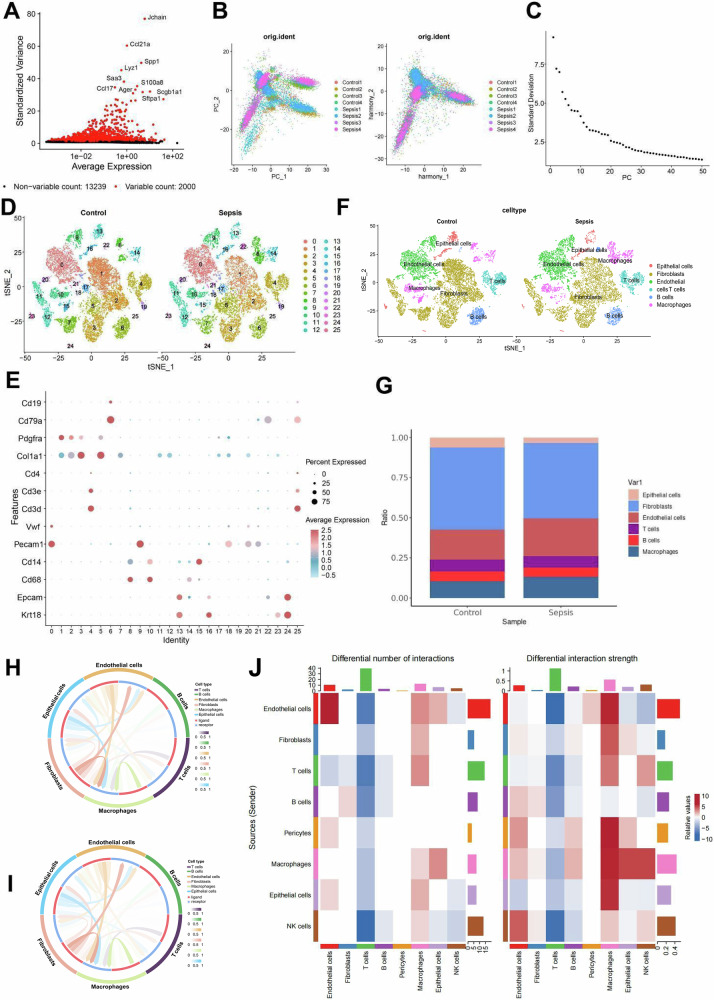
Analysis of changes in cell interactions under sepsis using single-cell data. **A** Variance analysis identifying highly variable genes, with red indicating the top 2000 highly variable genes and black indicating low variability genes; the top 10 highly variable genes are labeled; **B** Distribution of cells in PC_1 and PC_2 before batch correction (left) and after Harmony batch correction (right), with each point representing a cell; **C** Distribution of standard deviation for PCs, with important PCs showing higher standard deviation; **D** t-SNE clustering visualization displaying cell clusters in normal and sepsis samples, with each color representing a different cluster; **E** Expression of known lineage-specific marker genes in different clusters, with red indicating high average expression and blue indicating low average expression; **F** Cell annotation visualization based on t-SNE clustering, with each color representing a different subpopulation; **G** Bar plot showing the proportions of different cell types in lung tissue from normal and sepsis mice; **H** Intercellular interaction network in the normal group, with outer circle colors representing different cell types, inner circle red indicating cell receptors, blue indicating cell ligands, and the depth of line color indicating interaction strength; **I** Intercellular interaction network in the sepsis group, with outer circle colors representing different cell types, inner circle red indicating cell receptors, blue indicating cell ligands, and the depth of line color indicating interaction strength. **J** presents a heatmap illustrating the differences in the number and intensity of intercellular interactions between the sepsis and normal groups. The sample size for each group was *n* = 4.

Highly variable genes were identified through variance-based screening (Fig. 2A), and cell cycle phases were determined using the CellCycleScoring function (Fig. S1D). Principal component analysis (PCA) was then applied for linear dimensionality reduction, with heatmaps displaying the top genes contributing to PC_1 to PC_6 (Fig. S1E) and the spatial distribution of cells across PC_1 and PC_2 (Fig. 2B). To facilitate downstream analysis, batch correction was applied to the sample data using the Harmony package (Figs. S1F, 2B). Additionally, ElbowPlot was employed to rank the principal components (PCs) by standard deviation, showing that PC_1 to PC_20 adequately captured the information in the highly variable genes, providing meaningful differentiation of the cells (Fig. 2C).

Nonlinear dimensionality reduction was performed using the t-distributed stochastic neighbor embedding (t-SNE) algorithm based on the top 20 PCs to optimize clustering resolution (Fig. S1G). This analysis identified 26 clusters, each characterized by distinct gene expression profiles (Fig. 2D). Using known cell lineage-specific marker genes from relevant literature and the CellMarker online database [15], we annotated six cell types: T cells, B cells, epithelial cells, macrophages, endothelial cells, and fibroblasts (Fig. 2E, F). Specifically, clusters 4 and 25 corresponded to T cells; clusters 6 and 22 to B cells; clusters 13, 16, 23, and 24 to epithelial cells; clusters 8, 10, 14, and 15 to macrophages; clusters 0, 9, 18, 20, and 21 to endothelial cells; and clusters 1, 2, 3, 5, 11, 12, and 17 to fibroblasts.

Analyzing the cell proportions revealed that, compared to normal samples, the proportions of macrophages, endothelial cells, and T cells increased slightly in sepsis samples. This likely reflects an enhanced immune response to infection, with these cells playing critical roles in inflammation signaling, cell-mediated immunity, and vascular permeability regulation [16]. Conversely, the proportions of epithelial cells and fibroblasts decreased slightly, indicating potential epithelial barrier damage and impaired tissue repair processes due to the inflammatory environment, which affects both the function and quantity of these cells (Fig. 2G) [17].

We conducted a cell communication analysis to investigate the interactions between cells further. The results revealed markedly enhanced signaling between lung-resident macrophages and pulmonary endothelial cells in the sepsis group compared with controls (Fig. 2H–J). Macrophages release large amounts of inflammatory mediators, such as cytokines and chemokines, which activate pulmonary endothelial cells. Activated endothelial cells upregulate adhesion molecules and secrete inflammatory cytokines, thereby amplifying local inflammation, enhancing vascular permeability, promoting leukocyte infiltration, and aggravating lung tissue injury [18].

These findings indicate that interactions between pulmonary macrophages and endothelial cells are significantly enhanced under sepsis conditions.

### IL-1β as a potential key regulator in macrophage function during sepsis

**Fig. 3:**
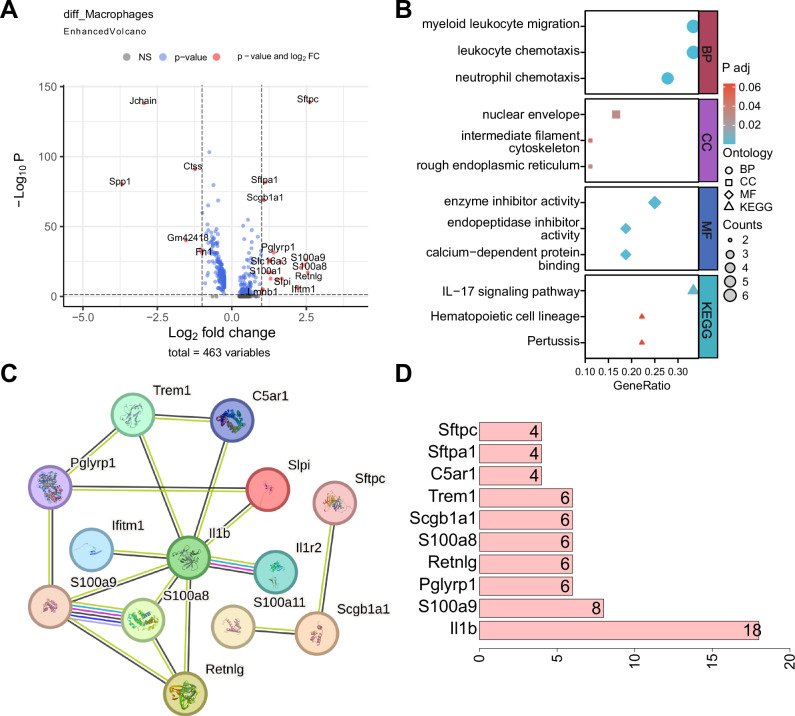
Identification of key regulatory molecules in macrophages during sepsis. **A** Volcano plot of DEGs in macrophages from normal versus sepsis groups; **B** GO and KEGG enrichment analysis of significantly upregulated genes; **C** PPI network of significantly upregulated genes, where connections indicate differentially expressed relationships; **D** Quantitative chart of connection nodes in the PPI network, with the x-axis representing the number of connection nodes and higher values indicating greater centrality. The sample size for each group is *n* = 4.

To identify key regulatory factors mediating macrophage involvement in sepsis, differential gene expression analysis was performed using the Seurat package. A total of 25 genes were differentially expressed between the sepsis and Control groups, including 19 upregulated and 6 downregulated genes (Fig. 3A). We focused on the significantly upregulated genes for further study. Enrichment analysis revealed functional changes in macrophages during sepsis. The upregulated genes, such as myeloid leukocyte migration, leukocyte chemotaxis, and neutrophil chemotaxis, were primarily enriched in BP. The cellular component (CC) pathways included the nuclear envelope, intermediate filament cytoskeleton, and rough endoplasmic reticulum. The molecular function (MF) processes were mainly enzyme inhibitor activity, endopeptidase inhibitor activity, and calcium-dependent protein binding. Kyoto Encyclopedia of Genes and Genomes (KEGG) pathway enrichment highlighted the IL-17 signaling pathway, hematopoietic cell lineage, and pertussis (Fig. 3B). These results indicate that macrophage functions related to immunity and inflammation undergo significant changes during sepsis.

We constructed a protein-protein interaction (PPI) network based on significantly upregulated genes to identify further vital regulatory molecules involved in macrophage activity during sepsis (Fig. 3C). Network centrality analysis identified IL-1β as the hub gene with the highest centrality score (Fig. 3D), indicating its pivotal role in coordinating macrophage-mediated inflammatory responses. IL-1β is a potent pro-inflammatory cytokine produced by activated macrophages and plays a role in initiating and regulating inflammatory responses. The significant increase in IL-1β expression in macrophages within the context of sepsis-induced lung injury indicates an exacerbated inflammatory response in lung tissue. While this may be a part of the body’s defense against infection, it can also lead to excessive inflammation and subsequent tissue damage.

These findings indicate IL-1β may be a critical regulatory molecule in macrophage function during sepsis.

### Sepsis induces metabolic reprogramming in pulmonary macrophages

**Fig. 4:**
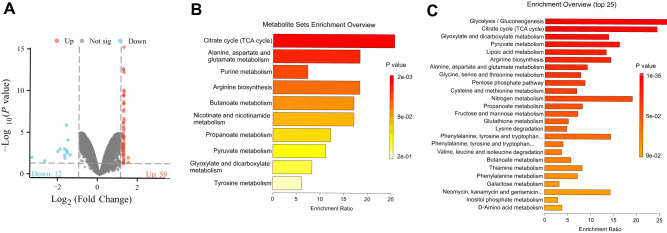
Metabolomic analysis of the impact of sepsis on pulmonary macrophages. **A** Volcano plot of differential metabolites, with the control group being the normal group; **B** Functional enrichment analysis of downregulated differential metabolites in the MetaboAnalyst database; **C** Functional enrichment analysis of upregulated differential metabolites in the MetaboAnalyst database. Sample size, *n* = 4.

Pulmonary macrophages from the Control and IL-1β^+^ groups were isolated from the lung tissues of sepsis mice and subjected to metabolomics analysis using LC-MS/MS. Comparative analysis identified significant alterations in 71 metabolites relative to controls, including 59 upregulated and 12 downregulated metabolites (Fig. 4A, Table S1). Metabolic pathway enrichment analysis using MetaboAnalyst showed that the downregulated metabolites were primarily enriched in the TCA cycle, pyruvate metabolism, and acetate and butyrate metabolism. In contrast, the upregulated metabolites were mainly associated with glycolysis/gluconeogenesis, the TCA cycle, and lactate metabolism (Fig. 4B, C).

The downregulation of the TCA cycle and pyruvate metabolism indicates that the energy metabolic pathways in lung-resident macrophages are inhibited under sepsis conditions. As a critical metabolite linking glycolysis and the TCA cycle, the reduced metabolism of pyruvate may lead to decreased production of acetyl-CoA, thereby affecting the efficiency of the TCA cycle. This reduction in acetyl-CoA can decrease the availability of substrates for the electron transport chain, impacting the OXPHOS process and potentially reducing ATP synthesis efficiency [19].

These results suggest that while metabolites related to OXPHOS are significantly reduced, those associated with aerobic glycolysis are markedly increased, highlighting the metabolic reprogramming of IL-1β^+^ macrophages under sepsis conditions. This metabolic shift emphasizes the importance of aerobic glycolysis in the inflammatory environment, providing energy and metabolic intermediates for macrophage function during sepsis.

In summary, sepsis induces significant enhancement of aerobic glycolysis in pulmonary macrophages.

### LPS-treated macrophages inhibit pulmonary endothelial cell proliferation and promote apoptosis

Based on the preceding bioinformatics analyses, IL-1β was hypothesized to serve as a pivotal regulatory molecule mediating macrophage-driven inflammation during sepsis. To investigate the regulatory role of IL-1β in macrophages on endothelial cells further, we first treated macrophages with LPS and then collected the conditioned medium to treat endothelial cells.

**Fig. 5:**
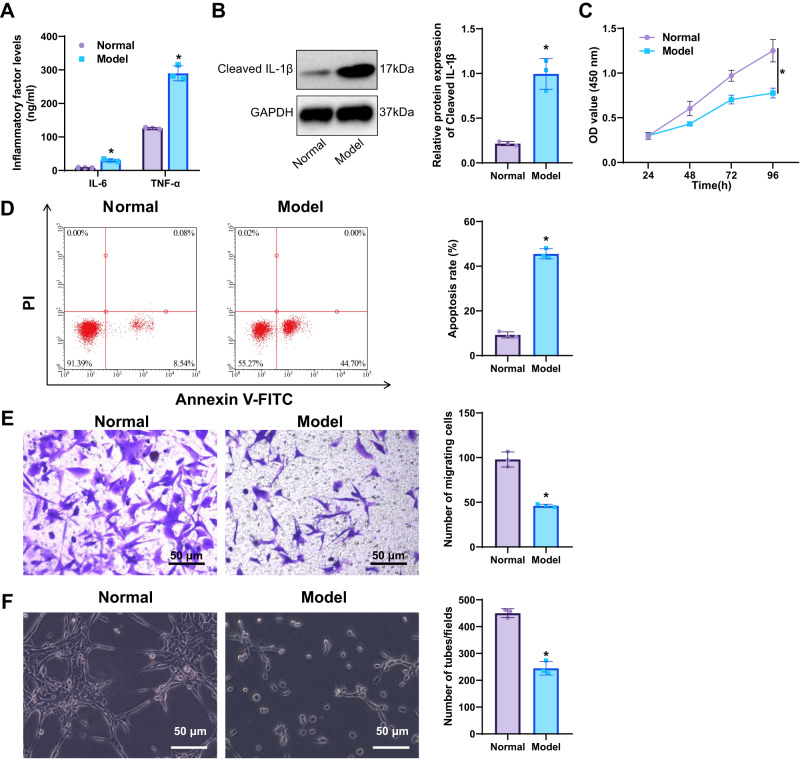
Impact of LPS-treated macrophages on the function of pulmonary endothelial cells. **A** ELISA measurement of IL-6 and TNF-α levels in the culture supernatant of LPS-treated macrophages; **B** WB analysis of IL-1β protein expression in LPS-treated macrophages; **C** CCK-8 assay measuring proliferation of endothelial cells; **D** Flow cytometry measuring apoptosis rates in endothelial cells; **E** Transwell assay measuring migration of endothelial cells (scale: 50 µm); **F** Tube formation assay measuring angiogenic capacity of endothelial cells (scale: 50 µm). *Indicates significance compared to the Normal group, *p* < 0.05, cellular experiments repeated thrice.

ELISA revealed that IL-6 and TNF-α levels in the supernatant of LPS-treated macrophage cultures were markedly elevated compared with the normal group (Fig. 5A). WB analysis was performed to detect IL-1β protein expression in LPS-treated macrophages, revealing a significant increase in IL-1β levels in the Model group compared to the Normal group (Fig. 5B). These findings confirm that LPS stimulation induces robust IL-1β expression and enhances the secretion of pro-inflammatory cytokines in macrophages.

The proliferation, apoptosis rate, migration, and tube formation ability of endothelial cells treated with conditioned medium from macrophages were assessed using CCK-8, flow cytometry, Transwell assays, and tube formation assays, respectively. CCK-8 results showed a significant reduction in the proliferation of endothelial cells treated with a conditioned medium from the Model group compared to the Normal group (Fig. 5C). Flow cytometry revealed a significant increase in apoptosis in endothelial cells treated with a conditioned medium from the Model group (Fig. 5D). Moreover, Transwell and tube formation assays showed pronounced inhibition of endothelial migration and tube-forming ability in the model group relative to the normal group (Fig. 5E, F). These results demonstrate that LPS-treated macrophages can inhibit endothelial cells’ proliferation, migration, and invasion capabilities in vitro while promoting their apoptosis.

### Silencing IL-1β reverses the inhibitory effects of LPS-treated macrophages on pulmonary endothelial cell proliferation and migration

**Fig. 6:**
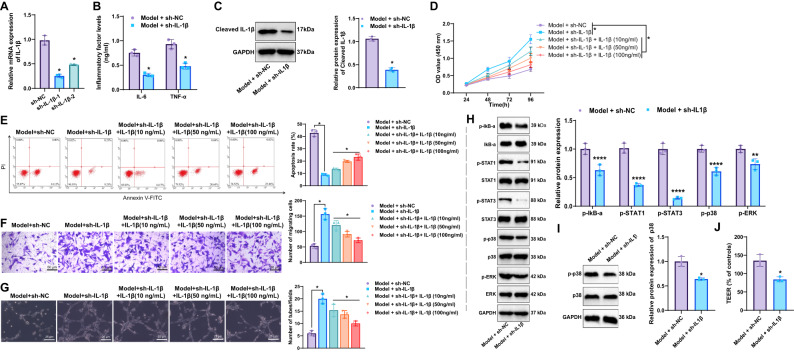
Silencing IL-1β affects the impact of LPS-treated macrophages on pulmonary endothelial cell function. **A** RT-qPCR validation of lentiviral shRNA silencing efficiency for IL-1β; **B** ELISA measurement of IL-6 and TNF-α levels in the culture supernatant of macrophages treated with sh-IL-1β and LPS; **C** WB analysis of IL-1β protein expression in macrophages treated with sh-IL-1β and LPS; **D** CCK-8 assay to assess the proliferation capacity of endothelial cells; **E** Flow cytometry to measure apoptosis rates in endothelial cells; **F** Transwell assay to evaluate migration capacity of endothelial cells (scale: 50 µm); **G** Tube formation assay to assess the tube formation capacity of endothelial cells (scale: 50 µm); **H** WB to detect the expression of endothelial cell proteins in each group. **I** shows Western blot analysis of p38 phosphorylation in macrophages treated with IL-1β and LPS. **J** shows TEER measurement of barrier integrity. *Indicates significance compared to the sh-NC or Model + sh-NC group, *p* < 0.05, cellular experiments repeated thrice.

The above in vitro experiments demonstrated that LPS-treated macrophages can inhibit endothelial cells’ proliferation, migration, and invasion capabilities while promoting their apoptosis. To examine the regulatory role of IL-1β in macrophages on endothelial cells, we silenced IL-1β in macrophages before treating them with LPS, then collected the conditioned medium to treat endothelial cells. The efficiency of IL-1β knockdown was confirmed by reverse transcription quantitative PCR (RT–qPCR), which showed that both sh-IL-1β-1 and sh-IL-1β-2 markedly reduced IL-1β expression compared with the negative Control (sh-NC). As sh-IL-1β-1 exhibited superior silencing efficiency, it was selected for subsequent experiments and designated as sh-IL-1β (Fig. 6A).

ELISA was used to measure the levels of IL-6 and TNF-α in the supernatant of LPS-treated macrophage cultures. The results showed that cytokine levels were markedly reduced in the Model + sh-IL-1β group compared with the Model + sh-NC group (Fig. 6B). WB analysis revealed that IL-1β protein expression was significantly decreased in the sh-IL-1β group compared to the Model group (Fig. 6C). These findings demonstrate that silencing IL-1β in macrophages can significantly suppress the secretion of inflammatory cytokines.

To evaluate the effects of IL-1β silencing in macrophages on endothelial cell function, proliferation, apoptosis, migration, and angiogenic capacity were assessed using CCK-8, flow cytometry, Transwell, and tube formation assays, respectively. CCK-8 results showed a significant enhancement in endothelial cell proliferation in the Model + sh-IL-1β group compared with the Model + sh-NC group (Fig. 6D). Flow cytometry revealed a significant decrease in apoptosis in endothelial cells treated with conditioned medium from the Model + sh-IL-1β group compared to the Model + sh-NC group (Fig. 6E). Consistently, Transwell and tube formation assays demonstrated increased migration and tube-forming abilities in endothelial cells exposed to conditioned medium from the Model + sh-IL-1β group relative to the Model + sh-NC group (Fig. 6F, G). Western blot analysis indicated that the p38 MAPK, NF-κB, and JAK/STAT signaling pathways were inhibited in endothelial cells treated with conditioned medium from the Model + sh-IL-1β group of macrophages (Fig. 6H). Collectively, these findings indicate that IL-1β knockdown in macrophages enhances endothelial proliferation, migration, and angiogenesis while reducing apoptosis, partially through suppression of the p38 MAPK pathway.

To verify whether macrophages directly secrete IL-1β to influence endothelial cells, endothelial cells treated with conditioned medium from the Model + sh-IL-1β group were supplemented with IL-1β (10 ng/mL, 50 ng/mL, 100 ng/mL for 24 hours). Compared to the Model + sh-IL-1β group, endothelial cells treated with IL-1β exhibited slightly reduced proliferation (Fig. 6D), migration (Fig. 6F), and tube formation abilities (Fig. 6G), along with a slight increase in apoptosis (Fig. 6E). These findings indicate that IL-1β secreted directly by macrophages does influence endothelial cells but has a limited impact. This finding suggests that the direct secretion of IL-1β is only one factor in how silencing IL-1β in macrophages enhances endothelial cell proliferation, migration, and invasion capabilities while reducing apoptosis. Endothelial cells were then treated with conditioned medium from IL-1β–treated macrophages. Western blot analysis demonstrated activation of the p38 MAPK pathway in endothelial cells exposed to the Model + IL-1β group (Fig. 6I). In addition, transepithelial electrical resistance (TEER) measurements confirmed impaired barrier function in this group (Fig. 6J). These findings indicate that IL-1β directly activates p38 MAPK in endothelial cells, thereby contributing to endothelial dysfunction.

### Knockdown of IL-1β in macrophages significantly alleviates sepsis-induced lung injury and inflammation

**Fig. 7:**
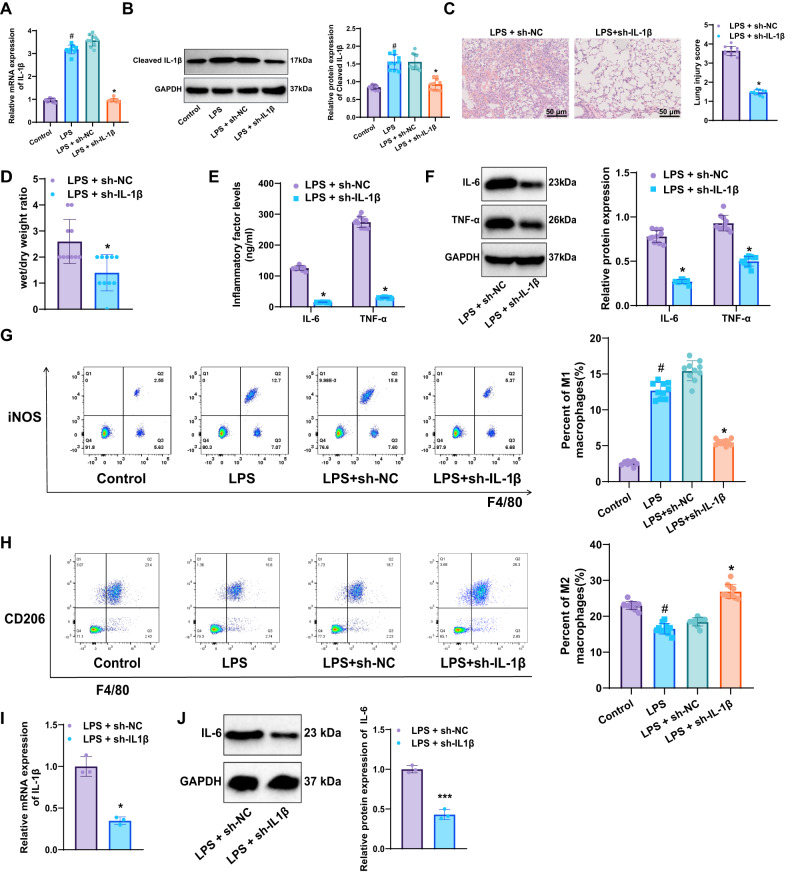
Impact of knocking down IL-1β on sepsis-induced lung injury and inflammatory response. **A** RT-qPCR analysis of IL-1β mRNA expression in lung tissue from different mouse groups; **B** WB analysis of IL-1β protein expression in lung tissue; **C** H&E staining to observe pathological changes in lung tissue from each group (scale: 50 µm); **D** Measurement of wet/dry weight ratios in lung tissue; **E** ELISA measurement of IL-6 and TNF-α levels in mouse serum; **F** WB analysis of IL-6 and TNF-α protein expression in lung tissue; **G** Flow cytometry to measure the proportion of M1 macrophages in lung tissue; **H** Flow cytometry to measure the proportion of M2 macrophages in lung tissue. **I** shows RT-qPCR detection of IL-1β mRNA expression in pulmonary macrophages isolated from each group. **J** shows Western blot analysis of IL-1β protein expression in pulmonary macrophages from each group. #Indicates significance compared to the Control group, *p* < 0.05, *indicates significance compared to the LPS + sh-NC group, *p* < 0.05, n = 10 mice per group.

In in vivo experiments, a sepsis mouse model with IL-1β knockdown was successfully established through the injection of recombinant adeno-associated virus (rAAV). The mice were divided into the Control, Sepsis model, and IL-1β knockdown Sepsis model. RT-qPCR and WB analyses confirmed a marked elevation of IL-1β mRNA and protein levels in the LPS group compared with controls, whereas both were significantly reduced in the LPS + sh-IL-1β group relative to the LPS + sh-NC group (Fig. 7A, B).

Histopathological examination of lung tissue by H&E staining revealed severe inflammatory cell infiltration, alveolar destruction, and interstitial edema in the LPS + sh-NC group, which were markedly alleviated following IL-1β knockdown (Fig. 7C). The wet/dry weight ratio of lung tissue was also measured, revealing a significant reduction in the LPS + sh-IL-1β group compared to the LPS + sh-NC group (Fig. 7D).

ELISA and WB analyses indicated that the levels of inflammatory markers IL-6 and TNF-α in both serum and lung tissue were significantly lower in the IL-1β knockdown group compared to the LPS + sh-NC group (Fig. 7E, F).

Flow cytometric analysis was performed to assess macrophage polarization in lung tissues. Compared with the Control group, the LPS group exhibited a markedly increased proportion of pro-inflammatory M1 macrophages and a concomitant decrease in anti-inflammatory M2 macrophages, indicating a shift toward a pro-inflammatory phenotype during sepsis. Notably, IL-1β knockdown significantly reversed this imbalance: the LPS + sh-IL-1β group displayed a reduced proportion of M1 macrophages and a corresponding increase in M2 macrophages relative to the LPS + sh-NC group (Fig. 7G, H). Pulmonary resident macrophages were isolated from lung tissues by flow cytometry, and their proteins were extracted for RT-qPCR and WB. The results showed that, compared with the LPS + sh-NC group, IL-1β mRNA and protein expression in macrophages from the lungs of the LPS + sh-IL-1β group were significantly reduced. These findings indicate that the lentiviral transduction efficiency in alveolar macrophages was satisfactory (Fig. 7I, J).

These findings suggest that silencing the IL-1β gene can significantly alleviate sepsis-induced lung injury and inflammation. Furthermore, it can regulate macrophage polarization, highlighting the crucial role of IL-1β in macrophage function during sepsis-induced lung injury.

## Discussion

Sepsis, a life-threatening systemic inflammatory response syndrome, frequently results in ALI, which is characterized by severe respiratory dysfunction and high morbidity [1, 2]. ALI severely compromises respiratory function, contributing to multi-organ failure and poor clinical outcomes [20, 21]. Previous studies have primarily investigated the contribution of inflammatory cytokines, macrophage activation, and endothelial dysfunction to the pathogenesis of sepsis-induced inflammation [22]. However, these studies have often relied on conventional biochemical methods that lack the resolution to elucidate intercellular interactions at the cellular level. This study employs scRNA-seq to provide a high-resolution perspective on the cellular dynamics and intercellular communication underlying sepsis-induced ALI, offering novel insights compared to traditional approaches.

The sepsis-induced lung injury model was validated by histological scoring and evaluation of the lung wet/dry ratio, while elevated inflammatory markers in lung tissue confirmed the presence of pulmonary inflammation. Through scRNA-seq analysis, we identified IL-1β^+^ lung-resident macrophages as key regulators in the pathogenesis of sepsis-induced lung injury. Previous studies have highlighted the pro-inflammatory role of IL-1β [23–25], but this study advances our understanding by detailing how IL-1β^+^ macrophages interact with pulmonary endothelial cells. These interactions occur through specific signaling pathways, leading to endothelial dysfunction, including impaired proliferation, reduced migration, and compromised barrier integrity. Notably, changes in IL-1β expression in macrophages not only influence direct cytokine signaling but also regulate macrophage differentiation and secondary cytokine secretion, which indirectly impacts endothelial cell functions [26]. This complex intercellular mechanism highlights the role of IL-1β in driving the inflammatory cascade and provides a rationale for considering IL-1β as a potential therapeutic target.

Further analysis revealed distinct behaviors of pulmonary endothelial cells under septic conditions, differing from conventional inflammatory models. Using CellChat, we demonstrated enhanced IL-1β-mediated communication between macrophages and endothelial cells, emphasizing the role of IL-1β in driving endothelial responses, including altered proliferation, migration, and tube formation capacities. This study delineates a positive feedback loop, where IL-1β^+^ macrophages activate endothelial cells, which in turn amplify inflammatory signaling, exacerbating lung tissue damage [7, 8]. Compared to tissue-level studies, our single-cell approach provides a more precise view of cell-type-specific interactions and their contributions to ALI pathophysiology.

On a metabolic level, macrophages exhibited significant metabolic reprogramming under septic conditions, as revealed by LC-MS/MS analysis. This reprogramming involves changes in several metabolic pathways that were previously underappreciated in conventional studies. These findings suggest that macrophages adapt to the highly inflammatory environment of sepsis by altering their metabolic states, potentially enhancing their pro-inflammatory functions. This new understanding of macrophage metabolism opens avenues for targeting metabolic pathways as a therapeutic strategy in sepsis-induced ALI.

To validate the role of IL-1β in lung injury, we conducted both in vitro and in vivo experiments. In vitro, silencing IL-1β enhanced endothelial cell proliferation and migration, contrary to its inhibitory effects observed in other inflammatory models. LPS-treated macrophages exhibited markedly increased IL-1β expression and robustly promoted the secretion of proinflammatory cytokines. Moreover, LPS-treated macrophages inhibited endothelial cell proliferation, migration, and invasion while promoting endothelial apoptosis. In vivo, IL-1β knockdown significantly alleviated lung injury in septic mice. These findings suggest that targeting IL-1β could be a viable strategy for mitigating lung damage. Interestingly, our exploratory experiments revealed that while direct IL-1β secretion from macrophages impacts endothelial cells, its effects are relatively limited. This indicates that IL-1β exerts its influence predominantly through indirect mechanisms, such as regulating macrophage differentiation or modulating the secretion of secondary cytokines. Additionally, a more complex signaling network involving multiple ligand-receptor interactions likely governs endothelial cell behavior during sepsis [27]. Future studies integrating transcriptomics and secretomics could further elucidate these mechanisms and their therapeutic potential.

This study also highlights the potential involvement of T cells in the pathogenesis of sepsis-induced lung injury. Despite the observed elevation in T cell abundance under septic conditions, the current work primarily focused on macrophage–endothelial cell interactions. T cells may contribute to lung injury through cytokine secretion and by modulating the activity of macrophages and endothelial cells. Future studies integrating single-cell transcriptomic analysis with functional validation are warranted to delineate T cell subpopulations and their cellular crosstalk, thereby achieving a more comprehensive understanding of their regulatory roles in sepsis-associated lung injury [5].

Current studies have highlighted that neutrophils, as key effector cells of the innate immune system, are rapidly recruited to lung tissue during the early stages of sepsis. By releasing neutrophil extracellular traps (NETs), reactive oxygen species, and various proteases, they directly mediate tissue injury and compromise barrier function [28, 29]. Importantly, activated macrophages further amplify neutrophil recruitment and activation by secreting IL-1β, TNF-α, and chemokines such as CXCL1 and CXCL2, thereby establishing a pro-inflammatory positive feedback loop [30, 31]. In addition, the dynamic balance of T-cell subsets—including Th1, Th17, and regulatory T cells—also plays a critical role in immune regulation during sepsis. For instance, IL-17 produced by Th17 cells enhances neutrophil recruitment and promotes macrophage production of IL-1β [32, 33], whereas Tregs may help maintain the balance between injury and repair by suppressing excessive inflammatory responses [34, 35]. Future investigations should integrate high-dimensional transcriptomic analyses with cell–cell interaction studies to systematically elucidate the spatiotemporal cooperative mechanisms of the macrophage–neutrophil–T-cell network in septic lung injury. Such insights could provide a foundation for developing combined therapeutic strategies that target multiple cell types and signaling pathways.

Although the proinflammatory role of IL-1β in sepsis-induced lung injury has been widely recognized [36, 37], this study is the first to define, at single-cell resolution, the specific subset of IL-1β⁺ lung-resident macrophages as drivers of pulmonary endothelial dysfunction. This advances beyond prior generalized descriptions of “macrophages” by uncovering subtype-specific regulatory mechanisms. Moreover, we integrated multi-omics approaches — scRNA-seq (transcriptomics), CellChat (cell–cell communication analysis), and LC-MS/MS (metabolomics). This integrative strategy not only validated known inflammatory signals but, more importantly, revealed the potential link between metabolic reprogramming of macrophages and their proinflammatory functions in sepsis, providing novel insight into immunometabolism in ALI and suggesting metabolic pathways as potential therapeutic targets.

While this study provides valuable insights, it is not without limitations. First, the mouse model, although mimicking aspects of human sepsis, may not fully represent human physiology and immune responses. Second, while scRNA-seq offers high cellular resolution, translating in vitro findings to in vivo applications remains complex. Additionally, the small sample size (four mice per group) may limit the generalizability of the results. Future studies should expand sample sizes, incorporate multi-center designs, and validate findings in human samples to strengthen translational applicability. Moreover, the dynamic progression of sepsis and its temporal mechanisms warrant further exploration using time-series analysis. As noted, we cannot exclude the systemic effects of IL-1β modulation, since other tissues may also participate in the observed pathological processes. The single-dose LPS model, while effective in mimicking Gram-negative sepsis–related ALI, cannot fully reproduce the clinical heterogeneity of human sepsis [38, 39]. Additionally, although RAW264.7 cells are widely used in macrophage studies, their metabolic state and functional responses may differ from those of primary lung-resident macrophages. Our experimental design primarily relied on the LPS model and cell line data without incorporating more clinically relevant systems, such as the cecal ligation and puncture (CLP) model or primary cell validation. The CLP model can better replicate the dynamic, polymicrobial nature of sepsis and would enhance the translational value of mechanistic studies. Future research should validate findings in multiple model systems (e.g., CLP, primary cell co-culture) and integrate multi-omics approaches to comprehensively evaluate host responses to different pathogens. Expanding sample size, performing temporal analyses, and including human tissue validation will also strengthen the reliability and clinical applicability of the findings.

Despite these limitations, this study significantly advances our understanding of the molecular interactions underlying sepsis-induced ALI. By identifying IL-1β^+^ macrophages and their communication with endothelial cells as pivotal drivers of lung injury, we demonstrate that sepsis involves a complex, multi-pathway inflammatory network in which IL-1β plays an important role. We highlight IL-1β signaling as a therapeutic target. Regulating IL-1β expression or its downstream pathways could reduce lung damage, providing a theoretical basis for developing small molecule inhibitors, neutralizing antibodies, or RNA-based therapies targeting IL-1β or related signaling pathways. Additionally, the methodologies employed in this study, such as scRNA-seq and intercellular communication analysis, serve as templates for studying other inflammatory diseases, potentially fostering innovation in therapeutic development.

**Fig. 8:**
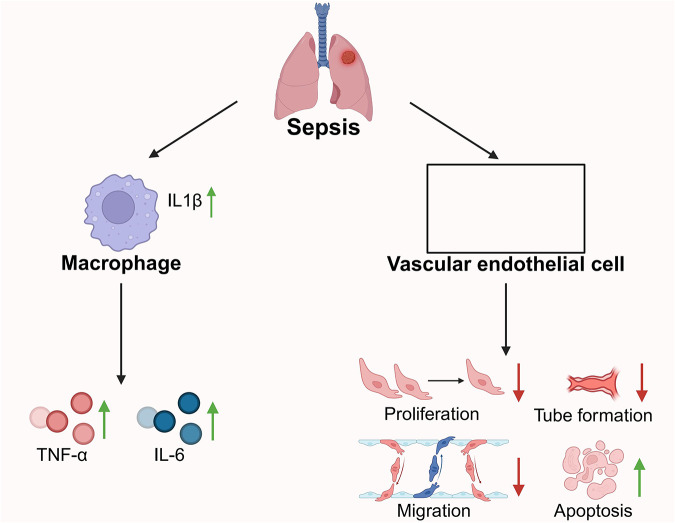
Molecular mechanism of interaction between IL-1β^+^ lung-resident macrophages and pulmonary endothelial cells in sepsis-induced lung injury.

This study explored the interaction mechanisms between IL-1β^+^ macrophages and pulmonary endothelial cells in sepsis-induced lung injury using scRNA-seq (Fig. 8). The results showed that sepsis significantly enhances signaling between IL-1β^+^ macrophages and endothelial cells, activating inflammatory responses and metabolic reprogramming, thereby exacerbating lung injury. In vitro experiments demonstrated that IL-1β^+^ macrophages inhibit endothelial cell proliferation and promote apoptosis, aggravating lung injury. In vivo experiments confirmed that IL-1β gene knockdown alleviates sepsis-induced lung injury and inflammation by regulating macrophage polarization, highlighting the critical role of IL-1β^+^ macrophages in sepsis-induced lung injury. This research provides new perspectives on the mechanisms underlying sepsis-induced lung injury and lays the foundation for developing therapeutic strategies. The experimental results of IL-1β gene knockdown support its clinical value as a therapeutic target. Future research should expand sample sizes and further investigate interactions between IL-1β^+^ macrophages and other immune cells to drive the clinical application of novel therapies.

## Materials and Methods

### Construction of sepsis mouse model

Male C57BL/6 mice (8–10 weeks old) were purchased from the Experimental Animal Center of Sun Yat-sen University and housed under standard conditions (ambient temperature 22 ± 2 °C, 12-hour light-dark cycle) with free access to food and water.

Sepsis was induced by intraperitoneal injection of lipopolysaccharide (LPS, 4 mg/kg, L5293, Sigma), while controls received sterile saline. Mice were randomly assigned to four groups (*n* = 10 per group): Control (saline injection), LPS (intraperitoneal injection of LPS), LPS + sh-NC (intraperitoneal injection of LPS + sh-NC treatment), and LPS + sh-IL-1β (intraperitoneal injection of LPS + sh-IL-1β treatment). Adenoviruses carrying sh-NC and sh-IL-1β (20 µl, 10^7^ particles/μL, Hanheng Biotechnology, Shanghai) were administered via tail vein injection one week prior to LPS administration [40]. Animals were euthanized 24 h after LPS injection, and serum and lung tissues were collected for analysis [41]. All procedures complied with the International Guidelines for the Care and Use of Laboratory Animals (https://arriveguidelines.org/arrive-guidelines/experimental-animals). Euthanasia was performed with sodium pentobarbital (150 mg/kg, intraperitoneally), and death was confirmed by cessation of respiration, heartbeat, and reflexes.

### **Lung wet/dry weight ratios**

After the removal of surface blood, lung tissues were immediately weighed to obtain the wet weight. Samples were dried at 70 °C for 48 h until constant weight, and the wet/dry ratio was calculated as (wet weight − dry weight) / dry weight to assess pulmonary edema.

### **H&E staining**

Lung tissues were fixed in 10% neutral buffered formalin (47608, Sigma-Aldrich, USA) for 24 hours, embedded in paraffin, and sectioned. Sections were dewaxed, rehydrated through a graded alcohol series, and stained with hematoxylin (#AR-0711, Dingguochangsheng Biotechnology) for 1–2 minutes, followed by eosin (#AR-0731, Dingguochangsheng Biotechnology). After dehydration, sections were mounted for observation. Lung injury was assessed using the American Thoracic Society’s blind scoring system, evaluating alveolar congestion, wall thickening, edema, and inflammatory cell infiltration.

### Flow Cytometry analysis

Single-cell suspensions were obtained from lung tissues following enzymatic digestion with collagenase I (SCR103, Sigma-Aldrich) and DNase I (260913, Millipore). Red blood cells were lysed using buffer (11814389001, Roche), and the remaining cells were centrifuged (500 × *g*, 5 min, 4 °C). Pellets were resuspended in 50 μL cell staining buffer (CSB, 420201, BioLegend, USA) and blocked with 10% FcR-Block (130-059-901, Miltenyi) for 15 min at 4 °C. Cells were then stained with antibodies against macrophage markers F4/80 (APC, 157305, BioLegend), iNOS (Alexa Fluor® 488, ab317527, Abcam), CD206 (FITC, 141703, BioLegend), and IL-1β (eFluor™ 450, 48-7114-82, eBioscience). After washing with CSB, samples were resuspended in 500 μL PBS and analyzed using a FACSAria Fusion flow cytometer (BD Biosciences).

### Lung tissue sample processing and single-cell suspension preparation

Lung tissues from normal and LPS-treated sepsis mice (*n* = 4 per group, 24 h post-treatment) were rinsed in ice-cold PBS. Tissues were minced using a 70 μm cell strainer (Falcon, USA) and digested with an enzyme mixture containing 1 mg/mL collagenase I (SCR103, Sigma-Aldrich) and 0.1 mg/mL DNase I (260913, Millipore) at 37 °C for 30 min with gentle agitation every 5 min. The digested material was filtered through a 40 μm strainer, treated with red blood cell lysis buffer (11814389001, Roche) for 5 min, washed with PBS, and resuspended in PBS containing 0.04% BSA. The final cell concentration was adjusted to 1000 cells/μL for downstream single-cell RNA sequencing.

### TEER measurement

TEER of cell monolayers was measured using an EVOM® volt-ohmmeter (World Precision Instruments, Aston, UK) equipped with STX-2 chopstick electrodes. Cells were seeded at 5 × 10⁵ cells/cm² on collagen-coated Transwell® inserts (0.4 μm pore size, 6.5 mm inner diameter; Corning Costar, Acton, MA, USA). After 24 h, the apical medium was removed to establish an air–liquid interface, and the basolateral medium was replaced every two days. Polarized monolayers cultured for 14 days were used for measurement following established protocols [42]. TEER values were expressed as percentages relative to the Control (baseline 520 Ω·cm²) and presented as mean ± SD. Statistical analysis was conducted using one-way ANOVA (*P < 0.05, **P < 0.01, ***P < 0.001). All experiments were independently repeated on at least three separate days with four replicates per condition (n = 4).

### scRNA-seq library construction and sequencing

Single-cell suspensions were processed using the Chromium platform (10x Genomics, USA), and libraries were prepared with the Chromium Single Cell 3′ Reagent Kit v3 according to the manufacturer’s instructions. Sequencing was performed on the Illumina NovaSeq 6000 system (Illumina, USA) with a target depth of approximately 50,000 reads per cell for each sample.

### t-SNE clustering analysis and cell communication

Dimensionality reduction of the scRNA-seq data was first performed by PCA on the top 2000 highly variable genes. The first 20 PCs, determined by the ElbowPlot function in Seurat, were used for clustering with the FindClusters function (resolution = 0.5). Nonlinear dimensionality reduction was carried out using the t-SNE algorithm. Cell subtypes were annotated based on marker gene expression and cross-referenced with the CellMarker database. Intercellular communication networks were analyzed using the CellCall package in R to infer ligand–receptor interactions among cell populations.

### Differential expression analysis

Differentially expressed genes (DEGs) in macrophages across treatment groups were identified using the FindMarkers function of the Seurat package in R. Genes with │logFC│ > 1 and *p* < 0.05 were considered significant. The results were visualized as volcano plots using the EnhancedVolcano package in R.

### Gene Ontology (GO) and KEGG enrichment analysis

Functional enrichment of DEGs was performed using the ClusterProfiler package in R, with *p* < 0.05 indicating significant enrichment. GO analysis covered BP, MF, and CC, while KEGG analysis identified key signaling pathways related to sepsis-associated upregulated genes. The KEGG results were visualized as bubble plots using ClusterProfiler.

### Construction of PPI networks

PPI analysis of significantly upregulated genes was conducted using the STRING database (http://string-db.org/), integrating experimental evidence, literature mining, and computational predictions. A confidence score threshold of 0.4 was applied. The interaction degree of each protein node was calculated and visualized in R, where higher node connectivity indicated greater centrality within the network.

### Metabolite extraction and LC-MS/MS analysis

Metabolites from IL-1β⁺ lung-resident macrophages were extracted using a prechilled 50% methanol–water solution (419193, Sigma-Aldrich, USA). Cell pellets were resuspended in 500 µL of extraction solvent, frozen at −80 °C for 15 min, and centrifuged at 12,000 *g* for 10 min at 4 °C. The supernatant was collected for LC-MS/MS analysis on a Thermo Fisher Ultimate 3000 LC system coupled to a Q Exactive mass spectrometer (Thermo Fisher Scientific, USA). Chromatographic separation was achieved on an Accucore C18 column (2.6 µm, 100 mm × 2.1 mm) using a gradient of 0.1% formic acid in water (phase A) and 0.1% formic acid in acetonitrile (phase B).

### Metabolic data processing and analysis

Metabolic data were processed using Compound Discoverer software (v3.1, Thermo Fisher Scientific, USA) for LC-MS/MS analyses. PCA and partial least squares discriminant analysis (PLS-DA) were employed to identify differential metabolites between groups. Metabolic pathway enrichment analysis was conducted via MetaboAnalyst (v5.0), with a significance threshold set at *p* < 0.05. This analysis specifically focused on the metabolic changes associated with oxidative phosphorylation (OXPHOS) and aerobic glycolysis.

### Cell infection and grouping

Mouse macrophage RAW264.7 cells (CBP60533, Nanjing Cobioer Biotechnology Co., Ltd.) were cultured in DMEM (11965118, Thermo Fisher Scientific, USA) supplemented with 10% fetal bovine serum (FBS, A5669701, Gibco, USA). Mouse microvascular endothelial cells (SNP-M001, Wuhan Sean Biotechnology Co., Ltd.) were maintained in DMEM containing 20% FBS. Both cell types were maintained at 37°C with 5% CO_2_ [41].

The lentiviral system was employed to silence IL-1β in RAW 264.7 macrophages. Sangon Biotech (Shanghai, China) provided plasmid construction and lentiviral packaging services. The sequences used were sh-NC: 5’-CGGTTGCTGGTTCGTTCTGGTG-3’, sh-IL-1β-1: 5’-CCGGGCAACCACTTACCTATTTATTCTCGAGAATAAATAGGTAAGTGGTTGCTTTTTG-3’, and sh-IL-1β-2: 5’-CCGGCAACAGTGGTCAGGACATAATCTCGAGATTATGTCCTGACCACTGTTGTTTTTG-3’. Plasmids were co-transfected with helper vectors into 293 T cells (CRL-3216, ATCC, USA). Following verification and purification, lentiviral particles were collected and used to infect RAW264.7 cells (MOI = 10, ~5 × 10⁶ TU/mL) in the presence of polybrene (5 µg/mL, TR-1003, Merck, USA). After 4 h, fresh medium was added, and the culture was replaced 24 h later. Transfection efficiency was evaluated at 48 h using a fluorescence reporter. Stable cell lines were established through puromycin selection (5 µg/mL, A1113803, Thermo Fisher Scientific, USA).

For LPS-induced modeling, macrophages were treated with 100 ng/mL LPS for 24 h. The macrophages were divided into four groups: Normal group (untreated macrophages); Model group (RAW264.7 macrophages treated with 100 ng/ml LPS for 24 hours); Model + sh-NC group (RAW264.7 macrophages infected with sh-NC lentivirus and treated with 100 ng/ml LPS for 24 hours); Model + sh-IL-1β group (RAW264.7 macrophages infected with sh-IL-1β lentivirus and treated with 100 ng/ml LPS for 24 hours). To further investigate the direct effect of IL-1β on endothelial cells, the conditioned medium from the Model + sh-IL-1β group was supplemented with recombinant IL-1β (10, 50, and 100 ng/mL). Macrophages and endothelial cells were co-cultured in Transwell plates for 24 h to establish an in vitro interaction system.

### ELISA detection of inflammatory cytokines

Supernatants from macrophage cultures and mouse serum were collected for cytokine quantification. IL-6 and TNF-α concentrations were measured using mouse ELISA kits (IL-6: ab222503; TNF-α: ab208348; Abcam, UK). Absorbance was read at 450 nm using a microplate reader (Bio-Rad Model 680, Bio-Rad, USA), and cytokine concentrations were calculated from standard curves.

### CCK8 assay for cell viability

Endothelial cells were seeded in 96-well plates at 3 × 10³ cells per well and co-cultured with macrophages for 24 h. The medium was then replaced with 100 µl of fresh medium containing 10 µl of CCK8 solution (HY-K0301, MCE). After incubating at 37 °C for 1.5 hours, absorbance was measured at 450 nm using a microplate reader (Bio-Tek, Winooski, VT, USA).

### Flow cytometry for apoptosis detection

Endothelial cells were harvested after 24 h of co-culture, washed twice with cold PBS, and resuspended in 1× Annexin V binding buffer. Apoptosis was evaluated using an Annexin V-FITC/PI Apoptosis Detection Kit (40302ES20, YEASEN, China). Cells were stained with 5 μL Annexin V-FITC and 5 μL PI for 15 min at room temperature (25 °C) in the dark and analyzed within 1 h using a flow cytometer (BD Biosciences, USA). Apoptotic cell populations were quantified based on Annexin V-FITC and PI fluorescence patterns.

### Cell migration assay

Endothelial cell migration was assessed using Transwell chambers (24-well format, 8 μm pore size). Cells from each group were seeded in the upper chamber with serum-free DMEM, while 600 μL of DMEM containing 15% FBS was added to the lower chamber. After incubation for 12 h, migrated cells on the lower membrane surface were fixed with 4% paraformaldehyde and stained with 10% crystal violet (HY-B0324A, MCE, USA) for 15 min. Cells in five random microscopic fields were counted under an inverted microscope (IX71, Olympus, Japan).

### Tube formation assay

The tube formation assay was conducted to assess the angiogenic capability of endothelial cells in vitro. Endothelial cells co-cultured for 24 hours were seeded at a density of 1 × 10^4^ cells per well into Matrigel-coated 96-well plates. The cells were cultured for 72 hours to observe tube formation. Tube formation was quantified by randomly selecting three fields of view and counting the number of tubes using an inverted microscope (Logos Biosystems, Villeneuve d’Ascq, France).

### RT-qPCR

Total RNA was isolated from cells using TRIzol reagent (15596026, Invitrogen, USA), and RNA concentration and purity were determined using a NanoDrop 2000 spectrophotometer (1011U, Nanodrop). RNA was reverse-transcribed into cDNA according to the PrimeScript RT Kit (RR047A, Takara) protocol, with reaction conditions set at 37 °C for 30–50 minutes and 85 °C for 5 seconds. RT-qPCR was performed using the Fast SYBR Green PCR Kit (RR820A, Takara) on an ABI PRISM 7300 system (Applied Biosystems). Each sample was run in triplicate, with GAPDH used as the internal control.

To ensure the accuracy of RT-qPCR results, the amplification efficiency and linearity of all primers were evaluated. Standard curve experiments were performed using a 10-fold serial dilution of template DNA to calculate the amplification efficiency (E) and correlation (R²) values. The amplification efficiency was calculated using the formula: Efficiency = (10^-1/slope^ - 1) × 100.

All primers demonstrated amplification efficiencies of 90%-110% with R² values exceeding 0.99, indicating good efficiency and linearity. Primer sequences, amplification efficiencies, and R² values are detailed in Table S2. Relative mRNA expression levels were quantified using the 2^^-ΔΔCt^ method. Experiments were performed in triplicate. All primers were synthesized by Sangon Biotech (Shanghai, China), with sequences provided in Table S2.

### Western Blot (WB) Analysis

Total protein was extracted from tissues using RIPA lysis buffer containing PMSF (P0013C, Beyotime, Shanghai, China). The samples were incubated on ice for 30 minutes, followed by centrifugation at 8000 *g* for 10 minutes at 4 °C to collect the supernatant. Protein concentration was determined using a BCA assay kit (23227, ThermoFisher, USA). Equal amounts of protein (50 μg) were mixed with 2 × SDS loading buffer, boiled at 100 °C for 5 min, and separated by SDS–PAGE. The proteins were then transferred onto a PVDF membrane (88518, ThermoFisher). The membrane was blocked with 5% BSA (9048-46-8, Sigma) at room temperature for 1 hour.

The PVDF membrane was incubated overnight at 4 °C with the following primary antibodies: rabbit anti-IL-1β (1:1000, ab315084, Abcam), IL-6 (1:1000, ab259341, Abcam), TNF-α (1:1000, ab183218, Abcam), p-p38 (1:1000, 9211, CST), p38 (1:1000, 9212, CST), p-ERK (1:1000, 4370, CST), ERK (1:1000, 4695, CST), and GAPDH (1:1000, ab9485, Abcam). After washing three times with TBST (10 minutes each), the membrane was incubated with HRP-conjugated secondary antibody (1:2000, ab97051, Abcam) for 1 hour. Following additional washes with TBST, the protein bands were visualized using an ECL detection kit (abs920, Absin).

Band intensities were quantified with Quantity One software (v4.6.2, Bio-Rad, USA). Relative protein expression was normalized to β-actin, and data were presented as mean ± standard deviation from three independent experiments.

Full and uncropped Western blot images are available in the supplementary materials.

### Statistical analysis

All data were obtained from at least three independent experiments and expressed as mean ± standard deviation (mean ± SD). Statistical analyses were performed using GraphPad Prism 9 (GraphPad Software, USA) and R software. Comparisons between two groups were analyzed using an unpaired Student’s *t*-test. For comparisons among three or more groups, one-way analysis of variance (ANOVA) followed by Tukey’s post hoc test was applied. When data did not meet the assumptions of normality or homogeneity of variance, the Mann–Whitney *U* test or Kruskal–Wallis *H* test was used instead. A two-tailed *p*-value < 0.05 was considered statistically significant.

## Supplementary information

Full and uncropped western blots

Supplementary figures and tables

## Data Availability

The datasets generated and/or analyzed during the current study are available in the manuscript and supplementary materials. Full and uncropped Western blot images are available in the supplementary materials. Further requests are available from the corresponding author on reasonable request.
