# Supplementary material for: IL-1β+ lung-resident macrophages mediate endothelial dysfunction and acute lung injury in sepsis through immune-metabolic crosstalk
Source: Cell Death Discov. 2025 Dec 8;12:85. doi: 10.1038/s41420-025-02868-0 (PMC12877074; doi:10.1038/s41420-025-02868-0)
Supplement: Supplementary file 1 — Full and uncropped western blots [file 41420_2025_2868_MOESM1_ESM.docx]

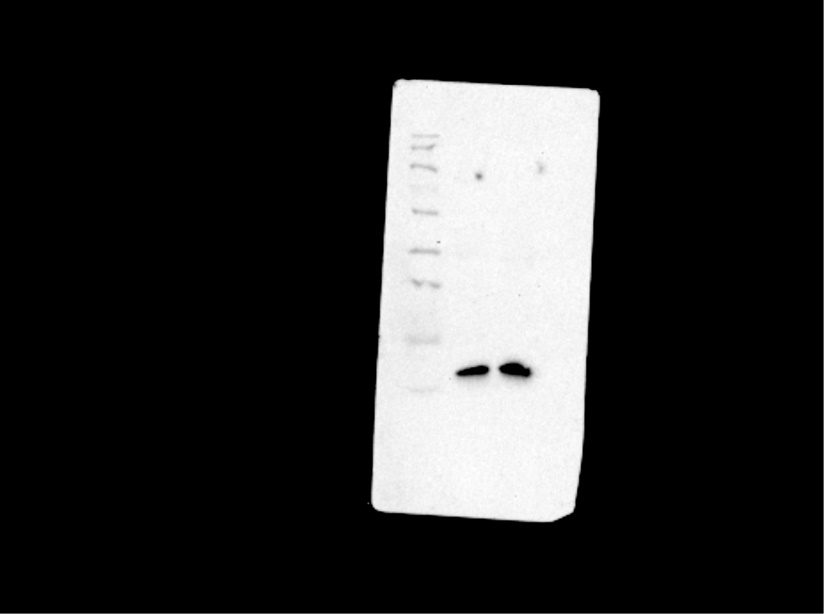


Figure 1D-1


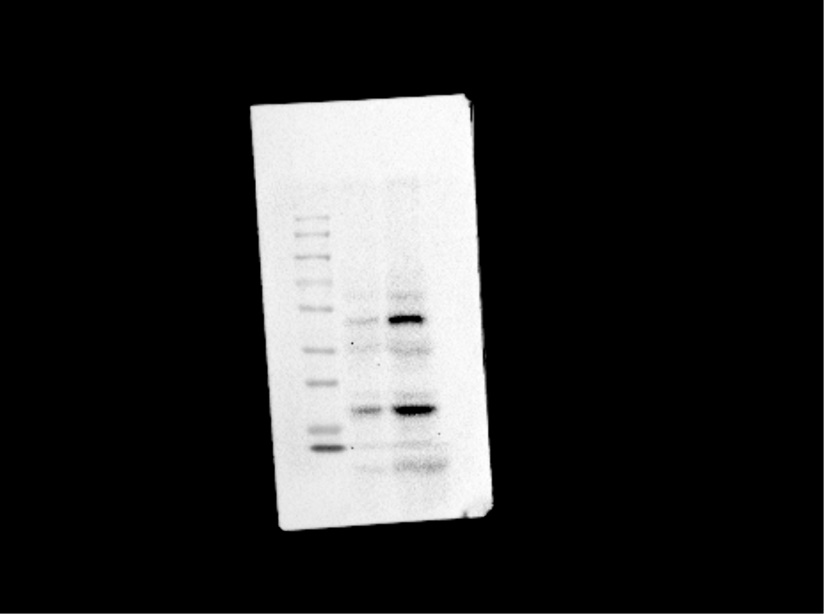


Figure 1D-2


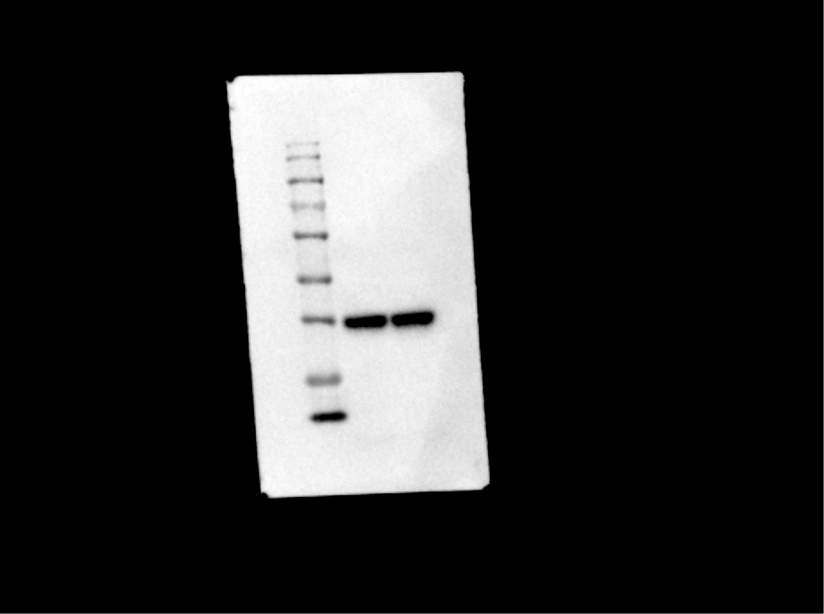


Figure 1D-3


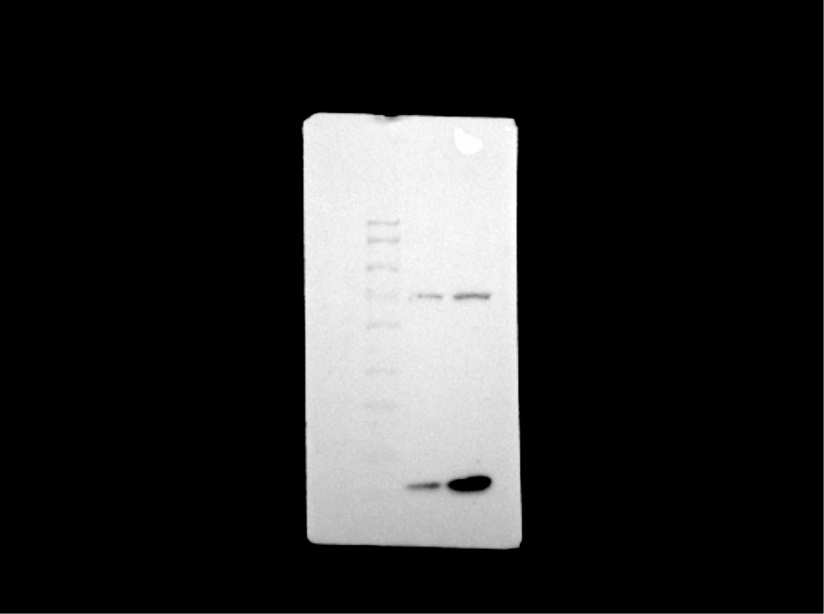


Figure 5B-1


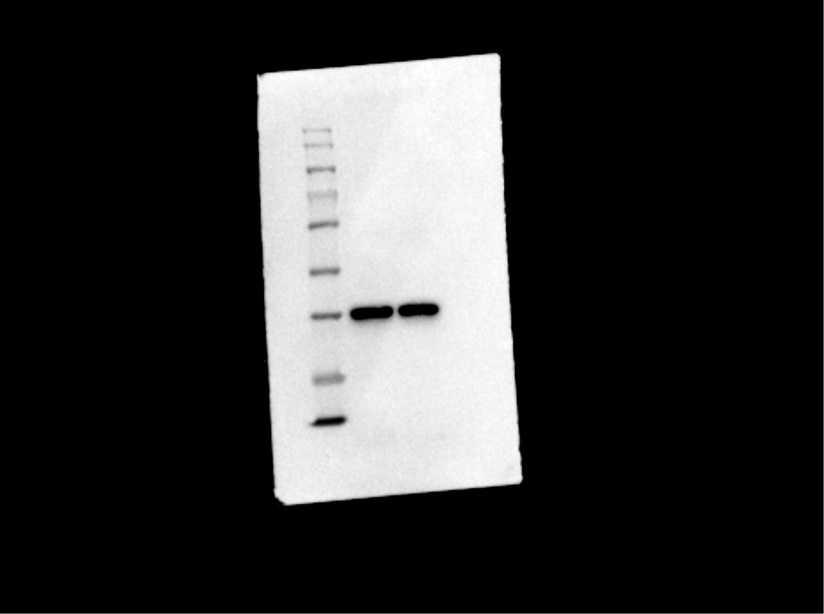


Figure 5B-2


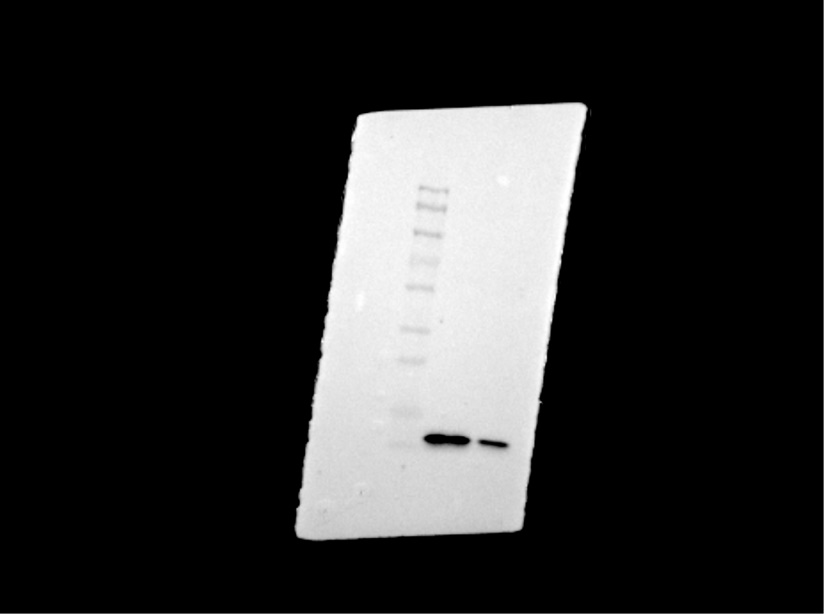


Figure 6C-1


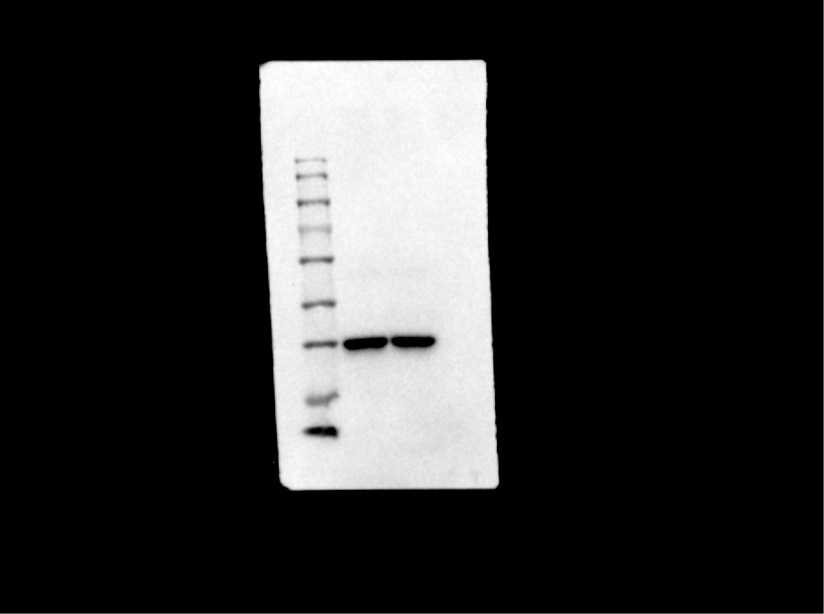


Figure 6C-2


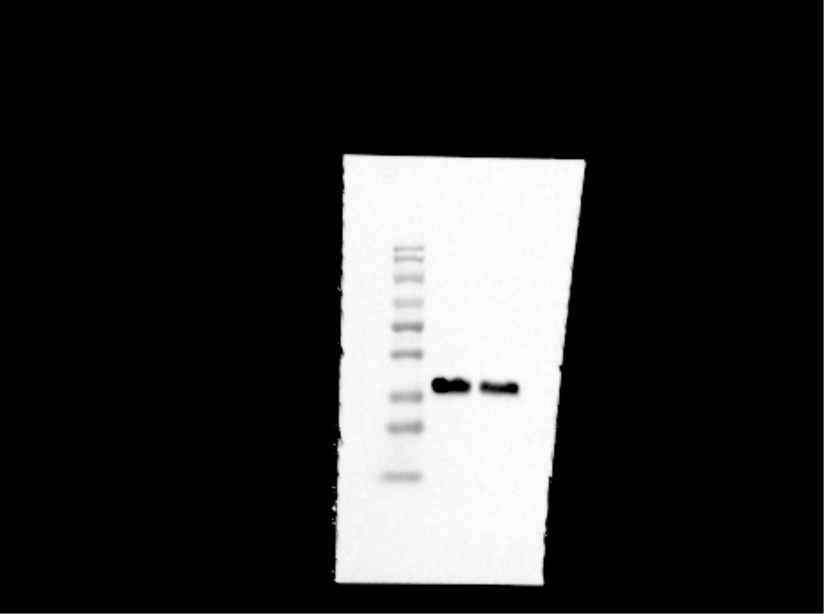


Figure 6H-1


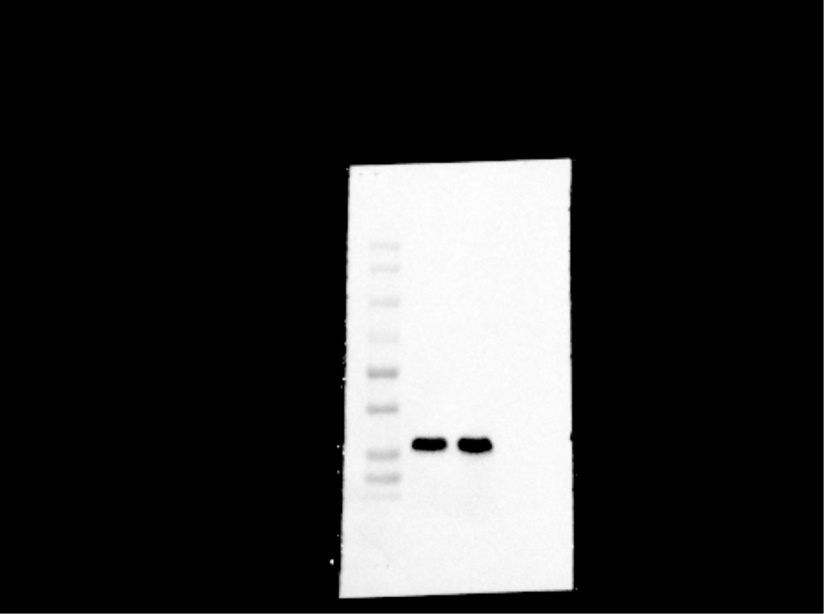


Figure 6H-2


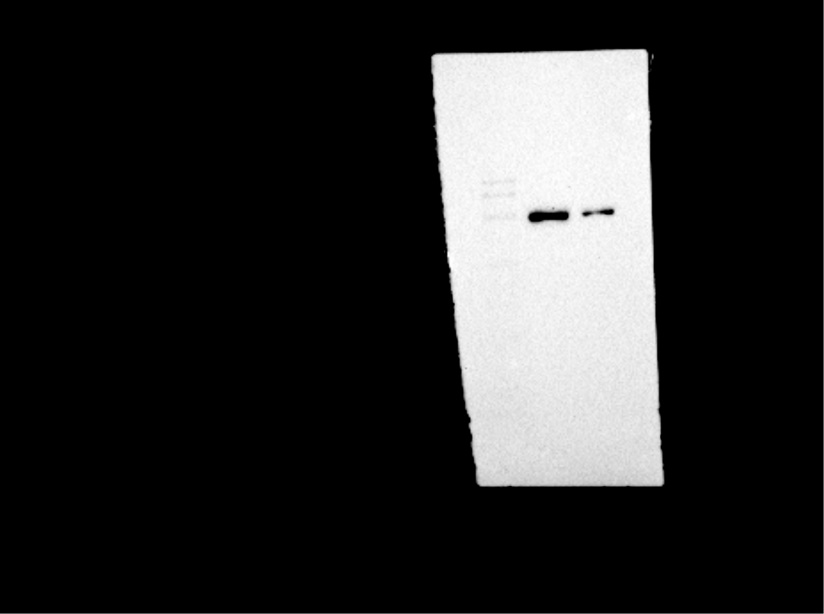


Figure 6H-3


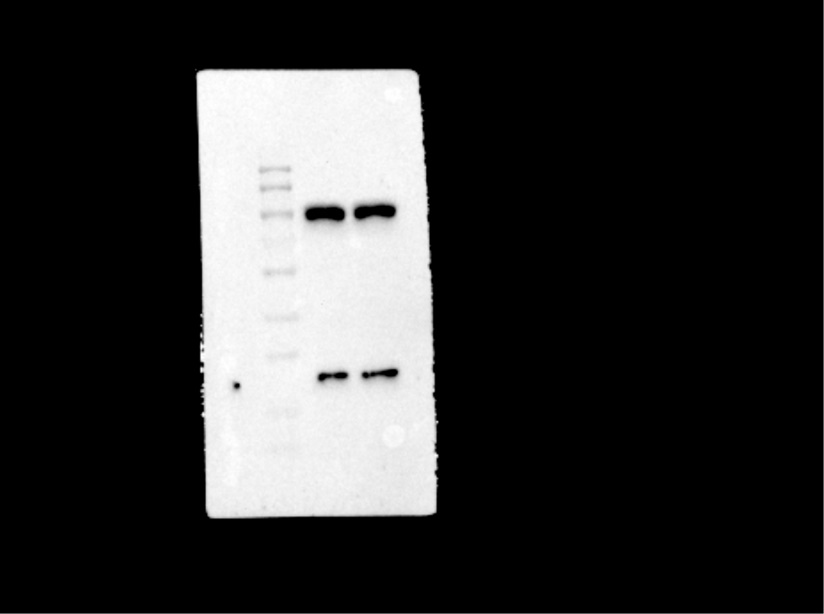


Figure 6H-4


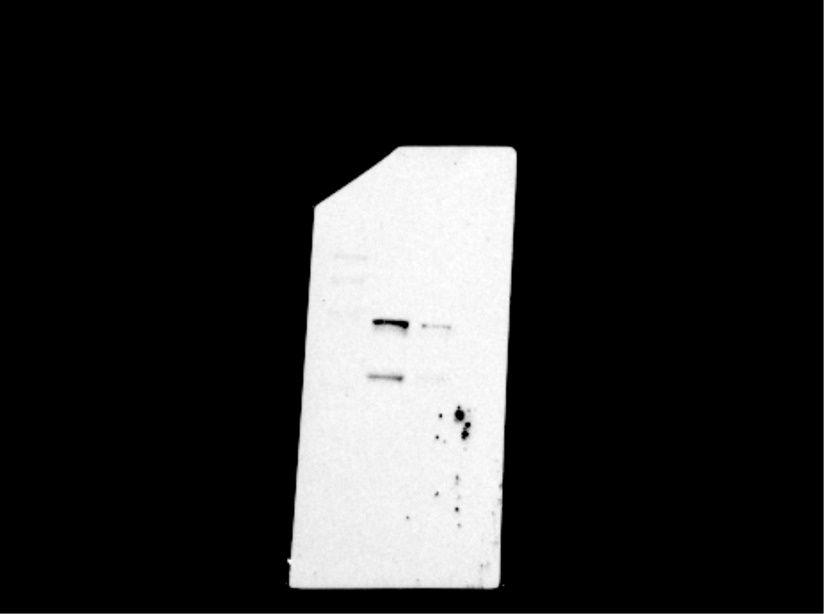


Figure 6H-5


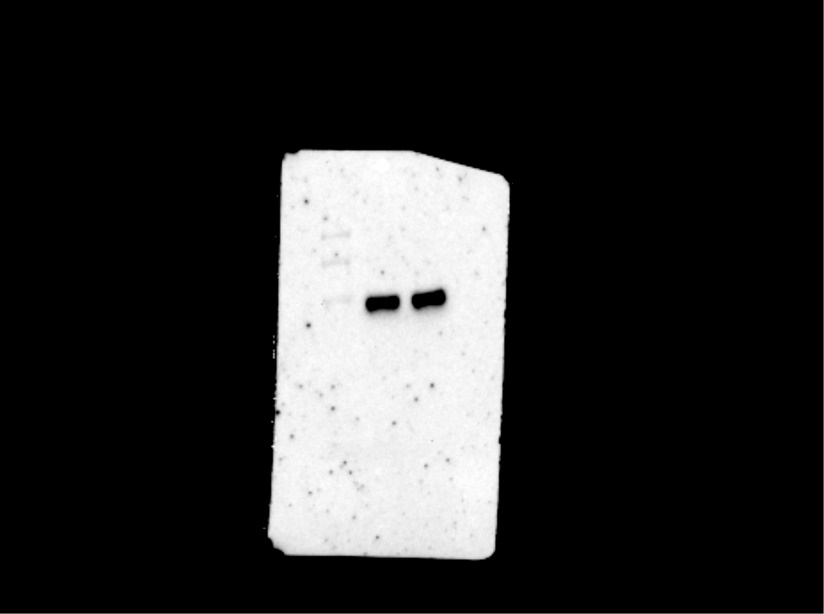


Figure 6H-6


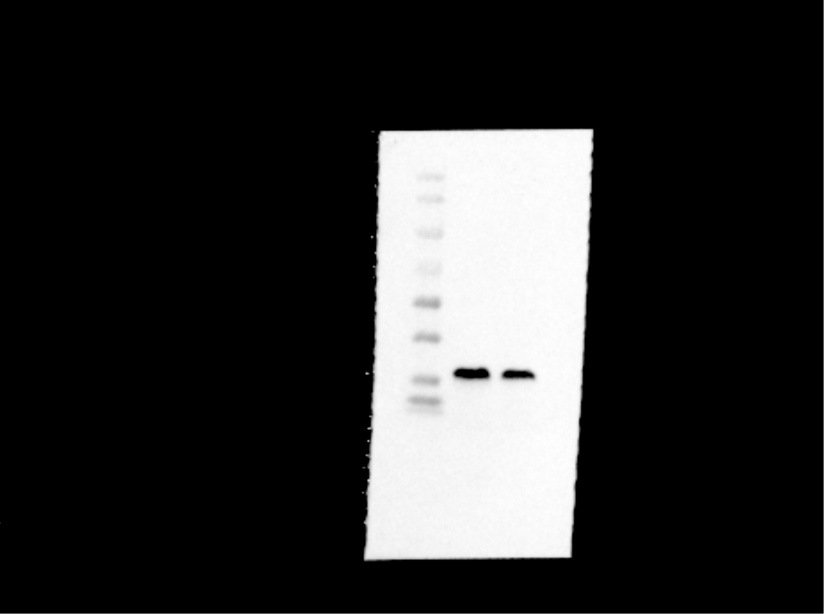


Figure 6I-1


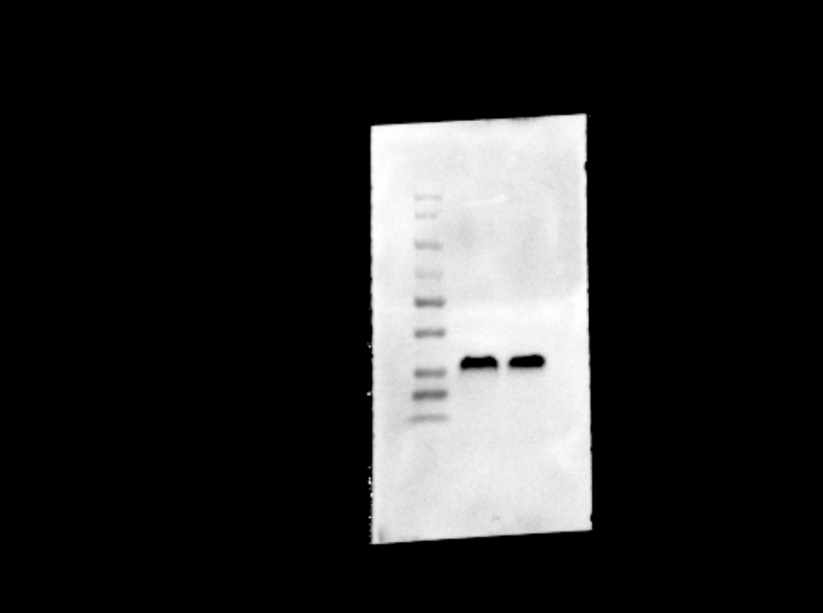


Figure 6I-2


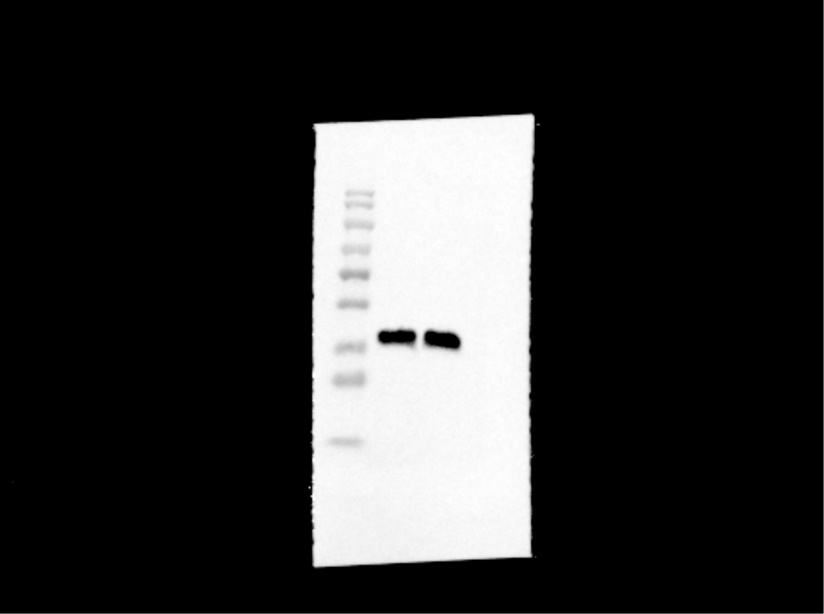


Figure 6I-3


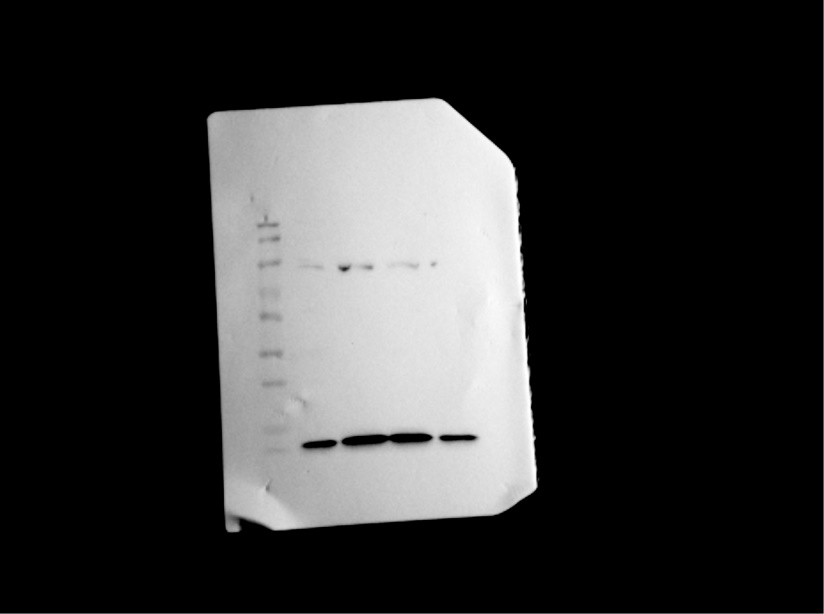


Figure 7B-1


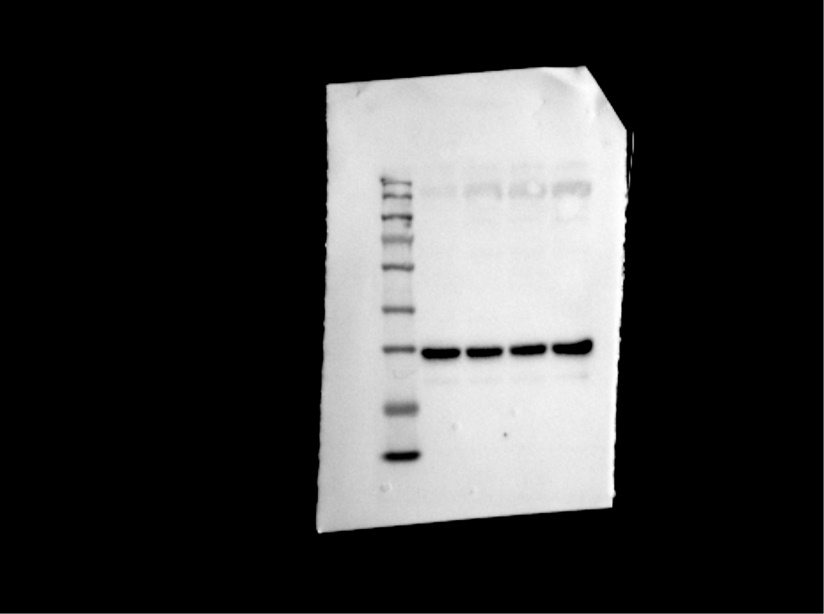


Figure 7B-2


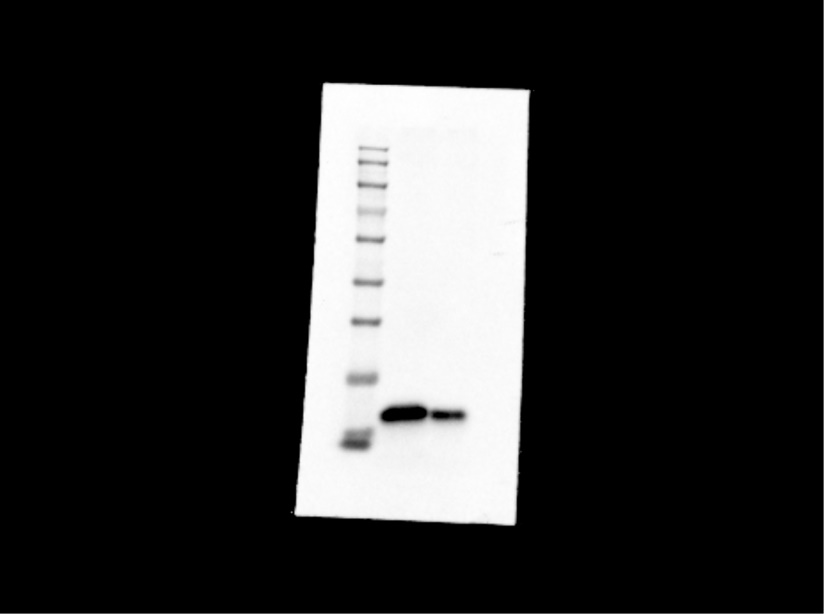


Figure 7F-1


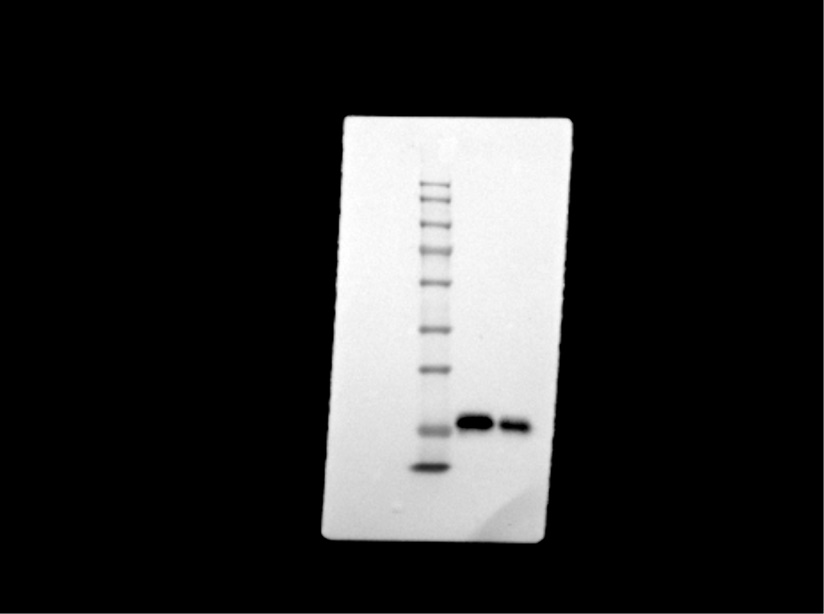


Figure 7F-2


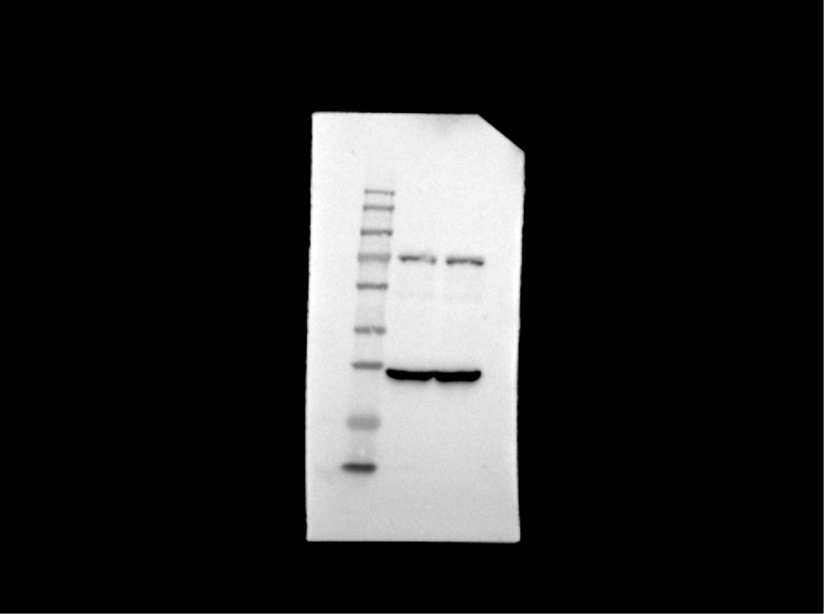


Figure 7F-3


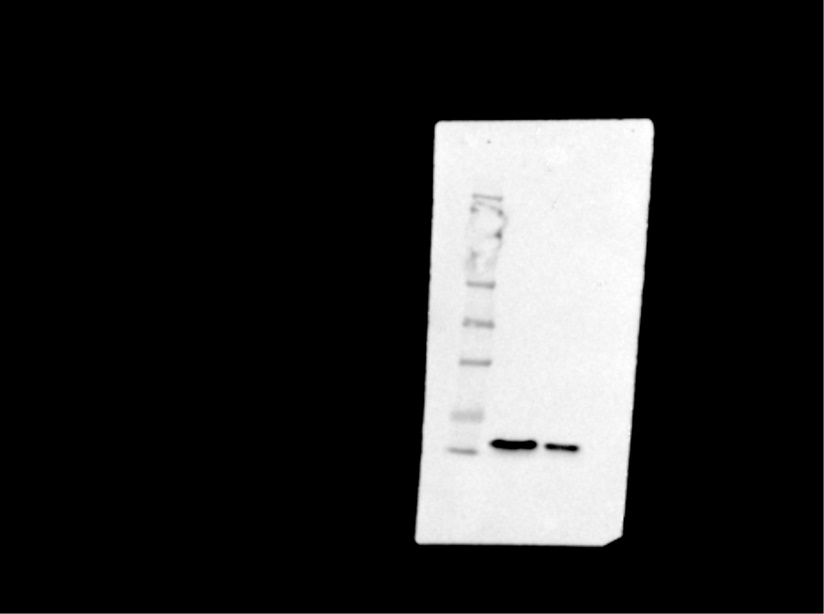


Figure 7J-1


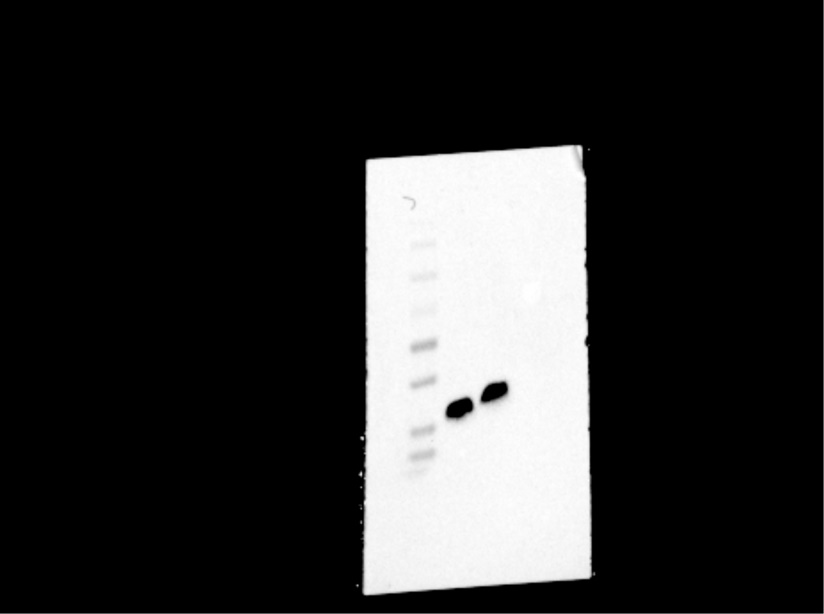


Figure 7J-2
