# Supplementary material for: IL-1β+ lung-resident macrophages mediate endothelial dysfunction and acute lung injury in sepsis through immune-metabolic crosstalk
Source: Cell Death Discov. 2025 Dec 8;12:85. doi: 10.1038/s41420-025-02868-0 (PMC12877074; doi:10.1038/s41420-025-02868-0)
Supplement: Supplementary file 2 — Supplementary figures and tables [file 41420_2025_2868_MOESM2_ESM.docx]

**Table S1. RT-qPCR primer sequence.**

| Gene | Sequence(5’-3’) | Increase efficiency (%) | Slope | R² value |
| --- | --- | --- | --- | --- |
| IL1b (mouse) | Forward: TGCCACCTTTTGACAGTGATG | 98.5 | -3.35 | 0.995 |
|  | Reverse: AAGGTCCACGGGAAAGACAC |  |  |  |
| GAPDH (mouse) | Forward: CCCTTAAGAGGGATGCTGCC  Reverse: TACGGCCAAATCCGTTCACA | 97.3 | -3.38 | 0.996 |

**Table S2. Differential expression of metabolites.**

| **gene** | **logFC** | **pvalue** | **ID** |
| --- | --- | --- | --- |
| C00001 | -3.249107328 | 0.011351495 | Water |
| C00002 | -2.626666758 | 0.029669518 | Adenosine triphosphate |
| C00003 | -2.003467888 | 0.002229947 | NAD |
| C00004 | -1.895352113 | 0.001157584 | NADH |
| C00007 | -1.681161966 | 0.001124547 | Oxygen |
| C00008 | -1.679417295 | 0.006099661 | ADP |
| C00009 | -1.654395364 | 0.013221661 | Phosphate |
| C00013 | -1.597397446 | 2.07887E-06 | Pyrophosphate |
| C00042 | -1.57341501 | 0.001907916 | Succinic acid |
| C00080 | -1.554688428 | 0.00309269 | Hydrogen Ion |
| C00122 | -1.525015216 | 0.000110142 | Fumaric acid |
| C00536 | -1.412750224 | 0.005482985 | Triphosphate |
| C00022 | 1.375866852 | 0.039298474 | Pyruvic acid |
| C00024 | 1.368364223 | 0.011159772 | Acetyl-CoA |
| C00031 | 1.159205512 | 6.10936E-07 | D-Glucose |
| C00033 | 1.158383681 | 2.77519E-10 | Acetic acid |
| C00036 | 1.157985677 | 3.2057E-08 | Oxalacetic acid |
| C00068 | 1.155881293 | 0.021042228 | Thiamine pyrophosphate |
| C00074 | 1.154996094 | 0.002298227 | Phosphoenolpyruvic acid |
| C00084 | 1.154659722 | 0.00554527 | Acetaldehyde |
| C00085 | 1.1526773 | 9.03357E-05 | Fructose 6-phosphate |
| C00103 | 1.152520182 | 0.00063876 | Glucose 1-phosphate |
| C00111 | 1.152101997 | 0.000276673 | Dihydroxyacetone phosphate |
| C00118 | 1.15191428 | 0.035253218 | Glyceraldehyde 3-phosphate |
| C00186 | 1.151774957 | 3.93768E-09 | Lactic acid |
| C00197 | 1.150054036 | 5.83088E-05 | D-Glycerate 3-phosphate |
| C00221 | 1.148921875 | 3.89012E-05 | beta-D-Glucose |
| C00236 | 1.148211806 | 4.6901E-06 | Glyceric acid 1,3-biphosphate |
| C00267 | 1.147888672 | 1.58915E-12 | alpha-D-Glucose |
| C00354 | 1.147301215 | 6.52332E-10 | Fructose 1,6-bisphosphate |
| C00469 | 1.14617513 | 3.34126E-07 | Ethanol |
| C00631 | 1.144730035 | 1.91659E-10 | 2-Phospho-D-glyceric acid |
| C00668 | 1.144591503 | 0.015691871 | alpha-D-Glucose 6-phosphate |
| C01159 | 1.142716146 | 8.01564E-06 | 2,3-Diphosphoglyceric acid |
| C01172 | 1.142365451 | 0.00112338 | beta-D-Glucose 6-phosphate |
| C01451 | 1.141365234 | 0.046821753 | Salicin |
| C05125 | 1.141282552 | 6.43724E-09 | 2-(a-Hydroxyethyl)thiamine diphosphate |
| C06186 | 1.140698351 | 0.000212349 | Arbutin |
| C06187 | 1.140084635 | 2.01672E-15 | Arbutin 6-phosphate |
| C06188 | 1.139996745 | 6.80215E-09 | Salicin 6-phosphate |
| C15972 | 1.137292318 | 0.046957852 | Enzyme N6-(lipoyl)lysine |
| C15973 | 1.136685113 | 0.000399744 | Enzyme N6-(dihydrolipoyl)lysine |
| C16255 | 1.13604579 | 3.61087E-05 | S-Acetyldihydrolipoamide-E |
| C16255 | 1.13471441 | 1.09334E-06 | S-Acetyldihydrolipoamide-E |
| C00022 | 1.134597873 | 0.000850125 | Pyruvic acid |
| C00024 | 1.134465495 | 0.048112942 | Acetyl-CoA |
| C00026 | 1.132853082 | 0.003836223 | Oxoglutaric acid |
| C00036 | 1.132171441 | 3.54501E-05 | Oxalacetic acid |
| C00042 | 1.130003906 | 0.000910813 | Succinic acid |
| C00068 | 1.129775391 | 4.92069E-08 | Thiamine pyrophosphate |
| C00074 | 1.129433594 | 8.2845E-10 | Phosphoenolpyruvic acid |
| C00091 | 1.12891276 | 0.005394501 | Succinyl-CoA |
| C00122 | 1.128603299 | 0.03615953 | Fumaric acid |
| C00149 | 1.127698134 | 4.72464E-05 | Malic acid |
| C00158 | 1.126814887 | 2.8921E-06 | Citric acid |
| C00311 | 1.126625651 | 6.6645E-10 | Isocitric acid |
| C00417 | 1.126245877 | 1.03822E-06 | cis-Aconitic acid |
| C05125 | 1.12605599 | 0.000368645 | 2-(a-Hydroxyethyl)thiamine diphosphate |
| C05379 | 1.126013238 | 0.000722184 | Oxalosuccinic acid |
| C05381 | 1.125567057 | 8.1877E-13 | 3-Carboxy-1-hydroxypropylthiamine diphosphate |
| C15972 | 1.124920139 | 5.05706E-05 | Enzyme N6-(lipoyl)lysine |
| C15973 | 1.123932509 | 0.007423658 | Enzyme N6-(dihydrolipoyl)lysine |
| C16254 | 1.122131944 | 6.50655E-13 | S-Succinyl- dihydrolipoamide-E |
| C16255 | 1.119668907 | 0.001778591 | S-Acetyldihydrolipoamide-E |
| C00011 | 1.118882469 | 1.93276E-05 | Carbon dioxide |
| C00014 | 1.091092244 | 0.000325515 | Ammonia |
| C00019 | 1.081229457 | 0.00085516 | S-Adenosylmethionine |
| C00021 | 1.076647231 | 0.00159268 | S-Adenosylhomocysteine |
| C00022 | 1.06965064 | 0.00047356 | Pyruvic acid |
| C00025 | 1.062695418 | 2.48232E-05 | Glutamic acid |
| C00026 | 1.038748885 | 1.98127E-05 | Oxoglutaric acid |

**
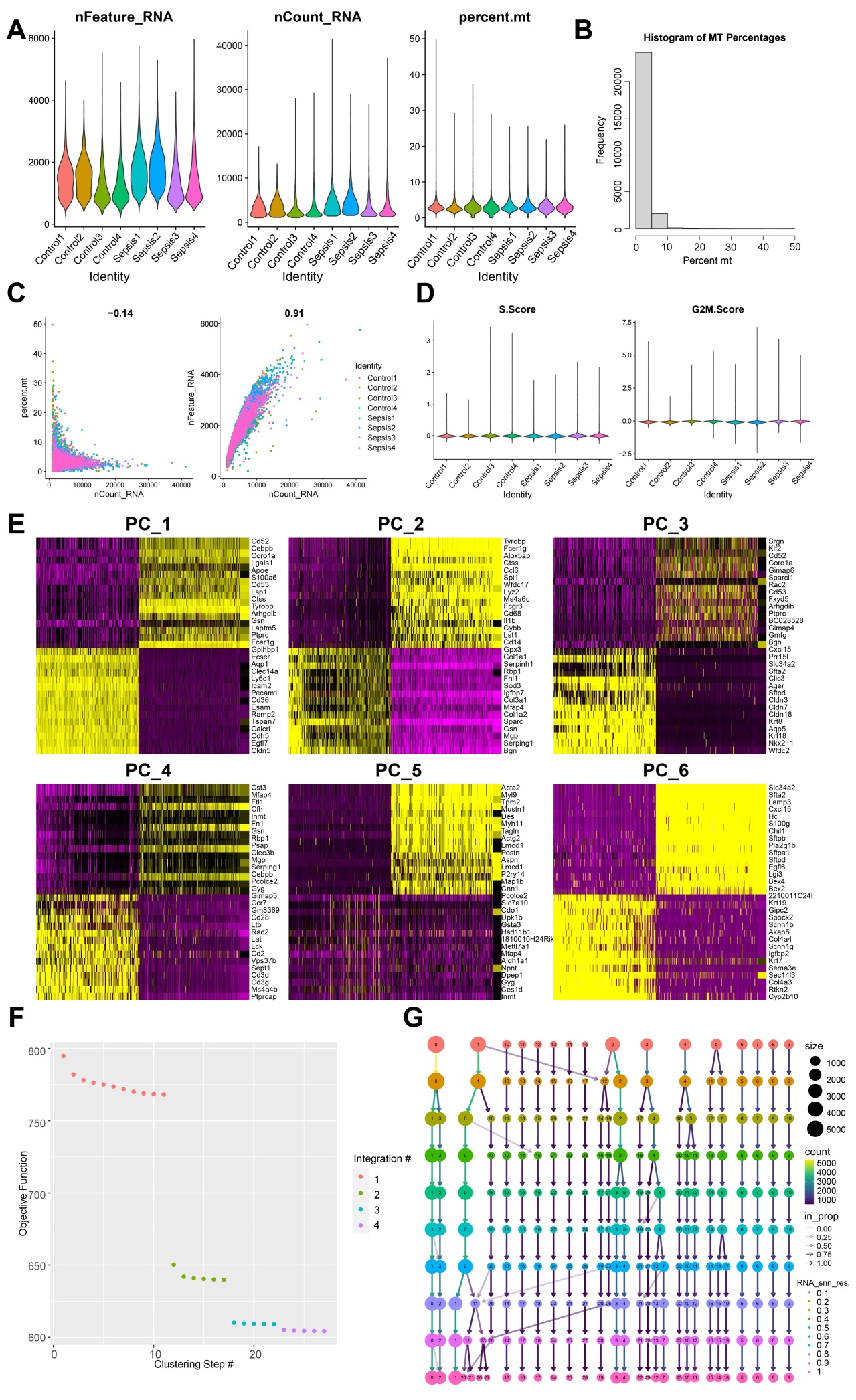
**

**Figure S1. Quality control and PCA dimensionality reduction in single-cell data.**

Note: (A) Violin plots showing the number of genes per cell (nFeature_RNA), the number of mRNA molecules (nCount_RNA), and the percentage of mitochondrial genes (percent.mt) in single-cell sequencing data; (B) Histogram of the distribution of different mitochondrial gene proportions in single-cell data; (C) Scatter plots showing the correlations between nCount_RNA and percent.mt, as well as nCount_RNA and nFeature_RNA, after data filtering; (D) Distribution of cell cycle phases; (E) Heatmap of the top 20 most highly correlated genes in PCA for PC_1 – PC_6, where yellow indicates upregulated expression and purple indicates downregulated expression; (F) Batch correction process by Harmony, with the x-axis representing the number of interactions; (G) Cell clustering based on different resolutions. Sample size n = 4.
